# Cross-sectional analysis of plasma and CSF metabolomic markers in Huntington’s disease for participants of varying functional disability: a pilot study

**DOI:** 10.1038/s41598-020-77526-9

**Published:** 2020-11-24

**Authors:** Andrew McGarry, John Gaughan, Cory Hackmyer, Jacqueline Lovett, Mohammed Khadeer, Hamza Shaikh, Basant Pradhan, Thomas N. Ferraro, Irving W. Wainer, Ruin Moaddel

**Affiliations:** 1 Department of Neurology, Cooper University Hospital and Cooper Medical School of Rowan University, Camden, NJ USA; 2 Biomedical Research Center, National Institute On Aging, National Institutes of Health, Baltimore, MD 21224 USA; 3 Department of Biomedical Sciences, Cooper Medical School of Rowan University, Camden, NJ, USA

**Keywords:** Analytical biochemistry, Mass spectrometry, Metabolomics, Biomarkers, Neurological disorders

## Abstract

Huntington’s Disease (HD) is a progressive, fatal neurodegenerative condition. While generally considered for its devastating neurological phenotype, disturbances in other organ systems and metabolic pathways outside the brain have attracted attention for possible relevance to HD pathology, potential as therapeutic targets, or use as biomarkers of progression. In addition, it is not established how metabolic changes in the HD brain correlate to progression across the full spectrum of early to late-stage disease. In this pilot study, we sought to explore the metabolic profile across manifest HD from early to advanced clinical staging through metabolomic analysis by mass spectrometry in plasma and cerebrospinal fluid (CSF). With disease progression, we observed nominally significant increases in plasma arginine, citrulline, and glycine, with decreases in total and d-serine, cholesterol esters, diacylglycerides, triacylglycerides, phosphatidylcholines, phosphatidylethanolamines, and sphingomyelins. In CSF, worsening disease was associated with nominally significant increases in NAD^+^, arginine, saturated long chain free fatty acids, diacylglycerides, triacylglycerides, and sphingomyelins. Notably, diacylglycerides and triacylglyceride species associated with clinical progression were different between plasma and CSF, suggesting different metabolic preferences for these compartments. Increasing NAD^+^ levels strongly correlating with disease progression was an unexpected finding. Our data suggest that defects in the urea cycle, glycine, and serine metabolism may be underrecognized in the progression HD pathology, and merit further study for possible therapeutic relevance.

## Introduction

Huntington’s Disease (HD) is a progressive, fatal neurodegenerative disorder characterized by motor, behavioral, and cognitive abnormalities. A polyglutamine expansion in the first intron of the gene coding for huntingtin (*htt*) promotes abnormal protein structure and subsequent disease pathophysiology^1^. The striatum and cortex are especially vulnerable, with both demonstrating significant histological abnormalities early in the course of the disease^1^. Whereas the central neurological effects of mutant huntingtin are well appreciated, mutant huntingtin (mhtt) is ubiquitous, with growing recognition of related abnormalities in leukocytes, muscle, hepatocytes, myelocytes, and cardiac function^2–7^. In parallel to the accumulating evidence suggesting HD is a disease of the body, a broad molecular reach for mutant huntingtin has also emerged: energetic dysfunction is suggested by elevated lactate in human HD cortical and striatal regions, abnormalities in Complex II, III, and IV of striatal mitochondria, and metabolic disturbances in glycolytic pathways, sphingolipids, redox signaling, and the tricarboxylic acid (TCA) cycle^8–13^. Phosphocreatine and sphingosine dysfunction are also observed in HD murine models^14,15^. These observations suggest the metabolome contains important information about disease pathophysiology across diverse regions and pathways in the body, and in turn possible novel targets for therapeutic approaches.

In an effort to characterize the metabolome, several studies have demonstrated catabolic changes considered as possible biomarkers of progression^16^. Among these, few consistent patterns have emerged, with discrepant data for amino acids^17–26^, catecholamine metabolites^27,28^, and lipids^23,29,30^. It is not well established how metabolomic changes correlate to clinical outcome measures over the spectrum of early to advanced manifest disease. Several factors have limited the successful identification of novel metabolomic biomarkers, including intra-individual variation, time of sampling, diversity in phenotype, assay reproducibility, and heterogeneity in disease stages assayed^16^. Several reports broadly define the populations under study (e.g., “pre-manifest” or manifest of varying severities), potentially contributing to inconsistency in the data by not more specifically defining disease stages against which metabolomic comparisons can be made. Changes of interest may be more clearly visible over time or understandable with a more structured measurement of progression, underpinning the importance of assessing early, mid-stage, and advanced disease as defined by a reliable and accepted indicator of clinical staging. With these aims, the present pilot study sought to identify correlations between domains of the Unified Huntington’s Disease Rating Scale (UHDRS) and metabolites across a spectrum of functional disability and disease severity, as defined by Total Functional Capacity (TFC) Score^31^. This was carried out using several metabolomic platforms (Biocrates p180 kit, ABSciex Lipidyzer platform, LC–MS/MS method for NAD^+^ and kynurenine) in both plasma and the corresponding CSF from participants with HD ranging from TFC Stage I (mild) to IV (severe).

## Results

Demographic information for participants is listed in Table 1. Ages of participants ranged from 24 to 60 years, with a mean age of 47.5 years. Nine of the 12 participants (75%) were female gender. Time since clinical diagnosis ranged from 1 to 12 years. CAG repeat lengths, when available (75% of participants) ranged from 41 to 50. Baseline TFC ranged from 3 to 13; three participants were HD Stage I (scores 12, 13, 13), four were Stage II (7, 8, 8, 9), four Stage III (4, 5, 6, 6), and one Stage IV (3). Concomitant medications are listed in Table 1.

**Table 1:** Participant demographic data.

Participant	Age at enrollment	Gender	Year of diagnosis	CAG expanded repeat length	Baseline TFC	HD stage	Concomitant medications
1	60	F	2009	41	8	II	Bupropion ER 300 mg QD Escitalopram 20 mg QD Donepezil 10 mg QD Alprazolam 5 mg PRN
2	55	M	2012	N/A	4	III	Sertraline 75 mg QD Lamotrigine 150 mg QD Tamsulosin 0.4 mg QD Deutetrabenazine 15 mg BID Quetiapine 25 mg HS Solifenacin 5 mg QD Senna 8.6 mg 2 tab BID
3	55	F	2014	43	7	II	Venlafaxine-XR 37.5 mg QD Loperamide 2 mg QD Mirtazapine 15 mg QD Diphenhydramine 25 mg PRN
4	45	F	2011	N/A	6	III	Tetrabenazine 25 mg BID Hydroxyzine 10 mg QD Amantadine HCl 100 mg QID Melatonin 10 mg HS
5	42	F	2014	N/A	3	IV	Citalopram 20 mg QD Amantadine 100 mg BID Risperidone 1 mg QD
6	60	M	2006	45	6	III	Tetrabenazine 12.5 mg BID Quetiapine 50 mg HS Venlafaxine-XR 150 mg Multivitamins with fluoride
7	52	F	2011	42	12	I	CoQ10, Ubiquinol, 200 mg Coenzyme Q10 100 mg QD Multivitamin QD Fish oil QD
8	46	M	2013	45	5	III	None
9	57	F	2015	43	8	II	None
10	30	F	2017	43	13	I	Lorazepam 1 mg QD Topiramate 25 mg QD Tizanidine 4 mg BID Sertraline 100 mg QD Meloxicam 15 mg QD Prazosin 1 mg QD Trazodone 50 mg HS
11	24	F	2015	50	13	I	None
12	44	F	2016	45	9	II	Citalopram 10 mg QD Levothyroxine 125 mcg QD

N/A: For CAG repeat length, diagnosis was established by first-degree relative and diagnostic confidence level IV.

**Figure 1:**
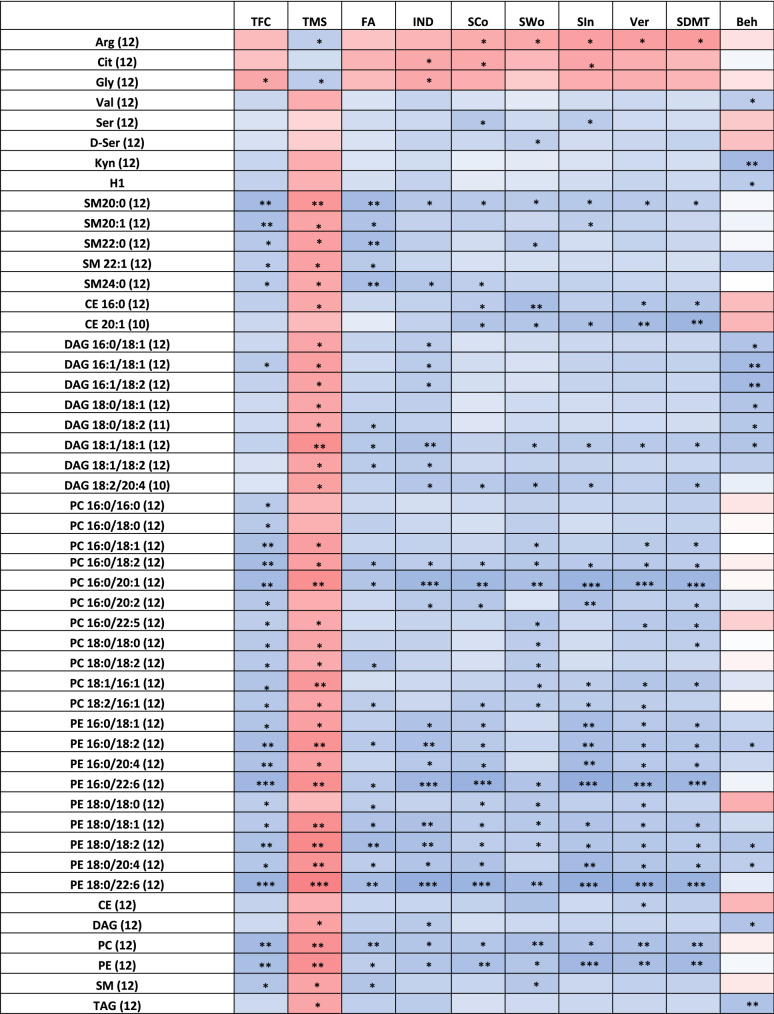
Correlations between metabolites in plasma and clinical outcomes. r-value correlations of − 0.7 to − 1.0 ( ) , − 0.4 to − 0.69 ( ) , 0.7 to 1.0 ( ) and 0.4–0.69 ( ) are shown; nominal *p* values represented as follows: *0.01 < *p* < 0.05; **0.001 < *p* < 0.01 and ***p < 0.001.

**Figure 2:**
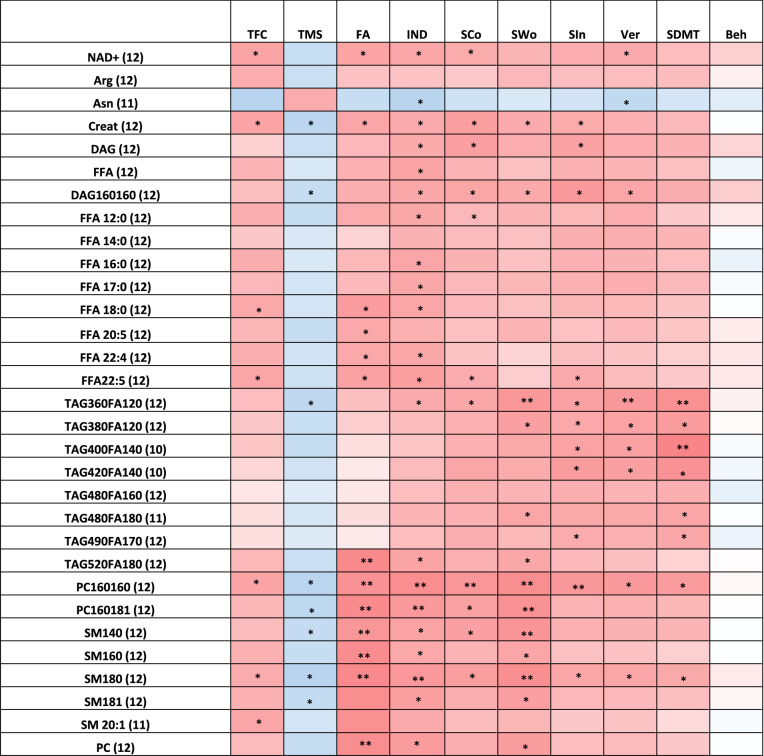
Correlations between metabolites in CSF and clinical outcomes. r-value correlations of − 0.7 to − 1.0 ( ), − 0.4 to − 0.69 ( ), 0.7 to 1.0 (  ) and 0.4–0.69 ( ) are shown; nominal *p* values are represented as follows: *0.01 < *p* < 0.05; **0.001 < *p* < 0.01 and ***p < 0.001.

UHDRS data for individual participants are listed in Table 2. Correlations between UHDRS domains and metabolites in plasma and CSF are depicted visually in Figs. 1 and 2, respectively, and numerically (including p-values) in Supplemental Tables 1 and 2.

**Table 2:** Participant unified Huntington’s disease rating scale values.

Subject	TFC	TMS	Functional assessment	Independence scale	Stroop Color naming	Stroop word reading	Stroop interference	Verbal fluency	SDMT	Behavioral assessment
1	8	19	20	80	56	69	32	32	30	41
2	4	63	4	55	15	23	15	13	24	10
3	7	52	19	65	25	25	13	8	7	25
4	6	64	13	65	12	7	8	3	0	26
5	3	81	5	50	6	9	11	2	3	19
6	6	56	15	75	36	27	18	13	6	6
7	12	14	24	95	75	100	41	45	50	0
8	5	34	22	80	40	69	25	18	23	23
9	8	47	23	85	49	63	28	29	26	2
10	13	7	25	100	57	68	43	44	46	50
11	13	14	25	100	100	73	59	46	52	18
12	9	39	20	70	32	52	24	20	18	10

### Plasma

Circulating levels of arginine correlated positively with the Total Motor Score (TMS) (*p* = 0.0435) and negatively with Stroop Color (*p* = 0.0482), Stroop Word (*p* = 0.0362), Stroop Interference (*p* = 0.0193), Verbal Fluency (*p* = 0.0169), and the Symbol Digit Modalities Test (SDMT) (*p* = 0.0121), suggesting higher levels of arginine associate with HD severity (Fig. 1, Supplemental Table 1). Citrulline, which is involved in the nitric oxide and urea cycles with arginine, also negatively correlated with the Independence Scale score (*p* = 0.0400), Stroop Color (*p* = 0.0368), Stroop Interference (*p* = 0.0290), and Verbal Fluency (*p* = 0.0568). Plasma glycine negatively correlated with TFC (*p* = 0.034), Independence Scale (IND) (*p* = 0.038), and nearly all cognitive measures while positively correlating to TMS (*p* = 0.039), suggesting that rising glycine levels correlate with broad clinical progression (Fig. 1; Supplemental Table 1). Total serine levels positively correlated with Stroop Color (*p* = 0.021) and Stroop Interference (*p* = 0.037), while d-serine (DSR) levels positively correlated with Stroop Word (*p* = 0.047) and trended towards positive correlation for the Independence Scale (*p* = 0.088) and SDMT (*p* = 0.081). Concentrations of DSR in plasma varied substantially (range 1.45 µM to 3.03 µM) with an average of 1.94 µM in plasma (Supplemental Table 3). Hexose demonstrated a positive trend with TFC (*p* = 0.073), IND (*p* = 0.0797), and a negative trend with TMS (*p* = 0.0676) suggesting lower levels with disease progression, though a contrasting positive correlation with Behavior scores (*p* = 0.037) was observed.

Circulating kynurenine levels in plasma showed positive correlation with Behavior scores (*p* = 0.0009), as did tryptophan (*p* = 0.001). While not nominally significant, increasing levels of kynurenine also negatively correlated with TMS (*p* = 0.0868). All other metabolites in the kynurenine metabolome showed no correlations with clinical outcomes, and the plasma kynurenine/tryptophan ratio was not associated with any measure of disease severity except a trending positive correlation with Behavior Scores (*p* = 0.06).

Circulating levels of several lipid classes decreased with clinical progression. These were cholesterol esters (CE), which positively correlated with Stroop Word (*p* = 0.0082) and Verbal (*p* = 0.0478); diacylglycerols (DAG), which negatively correlated with TMS (*p* = 0.0231) and positively with Independence (*p* = 0.0307) and Behavior (*p* = 0.0116); triacylglycerols (TAG), which negatively correlated with TMS (*p* = 0.0313) and positively with IND (*p* = 0.0357) and Behavior (*p* = 0.009); phosphatidylcholines (PC), which positively correlated with TFC (*p* = 0.0016), Functional Assessment (FA) (*p* = 0.0082), IND (*p* = 0.0165), Stroop color (*p* = 0.0196), Stroop Word (*p* = 0.0052), Stroop Interference (*p* = 0.0125), Verbal Fluency (*p* = 0.0081), SDMT (*p* = 0.0094) and negatively with TMS (*p* = 0.0045); phosphatidylethanolamines (PE), which positively correlated with TFC (*p* = 0.017), FA (*p* = 0.016), Stroop Word (*p* = 0.0217) and negatively with TMS (*p* = 0.0273); and sphingomyelin (SM), which displayed a negative correlation with TMS (*p* = 0.027) and a positive correlation with TFC (*p* = 0.017), FA (*p* = 0.0161) and Stroop Word (*p* = 0.022) (Fig. 1, Supplemental Table 1). No individual or total ceramide was found to correlate with any of the clinical outcomes. Among the CEs, only two species (CE 16:0 and CE 20:1) had a significant positive correlation with all cognitive components of the UHDRS (Fig. 1, Supplemental Table 1), with CE 16:0 also having a significant negative correlation with TMS (*p* = 0.0403). Eight diacylglycerols (DAGs) decreased with clinical progression, with all DAGs having a significant positive correlation with TMS (Fig. 1, Supplemental Table 1). DAG (18:1/18:1) and DAG (18:2/20:4) had a significant positive correlation with the majority of cognitive components of the UHDRS. Similarly, decreasing circulating levels of TAGs correlated with clinical progression; 92 TAG species had at least three components of the UHDRS that correlated significantly (Supplemental Fig. 1). Eleven PCs associated with clinical progression, with PC (16:0/18:2) and PC (16:0/20:1) having a significant correlation with nearly all UHDRS components (Fig. 1, Supplemental Table 1). Circulating concentrations of nine PEs decreased with clinical progression, with PE (18:0/18:2) having significant correlation with all 10 measured outcomes, and PE (16:0/18:2), PE(16:0/22:6), PE (18:0/18:1), PE (18:0/20:4), and PE (18:0/22:6) having a significant correlation with 9 of the 10 measured outcomes. Of all measured SMs, SM 20:0, SM 20:1, SM 22:0, SM 22:1 and SM 24:0 positively correlated with clinical outcomes. SM 20:0 positively correlated with all cognitive components of the UHDRS, and SM 20:1 was also significant or trending for the majority of the cognitive components.

### CSF

Among amino acids in CSF, only arginine and asparagine correlated with clinical outcomes. Arginine negatively correlated with moderate strength to TFC (*p* = 0.053) and trended positively with TMS (*p* = 0.07) and Stroop Word (*p* = 0.099), suggesting higher levels of arginine are associated with worsening disease (Fig. 2; Supplemental Table 2). Asparagine had a significant positive correlation with TFC (*p* = 0.013), IND (*p* = 0.016) and Verbal Fluency (*p* = 0.044), while negatively correlating with TMS (*p* = 0.0602). CSF concentrations of creatinine correlated with most of the outcome measures, showing a negative correlation with TFC (*p* = 0.0272), FA (*p* = 0.0324), IND (*p* = 0.0431), Stroop Color (*p* = 0.0155), Stroop Word (*p* = 0.0422), Stroop Interference (*p* = 0.0481) and positive with TMS (*p* = 0.0134). d-Serine measured in the CSF was not significantly correlated with any individual UHDRS domain. Glycine levels across all 12 participants were near or below the limit of quantitation; however, those with more severe TFC scores had relatively higher circulating levels (Supplementary Table 4).

No CSF kynurenine metabolites had a significant correlation with clinical outcome measures. Circulating kynurenic acid levels trended negatively with Stroop Word and SDMT outcomes (*p* = 0.0994 and 0.0774, respectively). Similar trends were observed for the kynurenic acid/kynurenine ratio with SDMT (*p* = 0.0981). The kynurenine/tryptophan ratio in the CSF was not associated with change in any UHDRS domain.

The CSF NAD^+^ metabolome demonstrated low concentrations (9 to 30 nM) (Supplemental Table 4), similar to previous reports; this may represent contamination from a small number of cells that underwent cytolysis^32^. Circulating levels of NAD^+^ negatively correlated with TFC (*p* = 0.0271), FA (*p* = 0.0281), IND (*p* = 0.0312), Stroop Color (*p* = 0.0450), Stroop Word (*p* = 0.0822), Stroop Interference (*p* = 0.0546) and Verbal Fluency (*p* = 0.0438), and had a positive trend with TMS (*p* = 0.0623) (Fig. 2, Supplemental Table 2), suggesting increasing levels of NAD^+^ in the CSF correlate with disease progression.

Free fatty acids (FFA) showed negative correlation trends for Stroop Color (*p* = 0.08), Interference (*p* = 0.067), Verbal Fluency (*p* = 0.089), FA (*p* = 0.067), and TFC (*p* = 0.082) and significant negative correlation with IND (*p* = 0.027) (Fig. 2; Supplemental Table 2). Increasing circulating levels of several saturated long chain saturated fatty acids (LCFAs) correlated significantly with clinical progression: stearic acid (C18:0), which correlated significantly with TFC (*p* = 0.037), FA (*p* = 0.014) and IND (*p* = 0.029) and trended for Stroop Color (*p* = 0.11), Interference (*p* = 0.09) and Verbal Fluency (*p* = 0.106); margaric acid (C17:0), which negatively correlated with IND (*p* = 0.034) and Verbal Fluency (*p* = 0.054) and trended negatively with TFC (*p* = 0.10), Stroop Color (*p* = 0.099), Stroop Word (*p* = 0.097), and Interference (*p* = 0.065); Palmitic Acid (C16:0), with significant negative correlation for IND (*p* = 0.029) and negative trend with TFC (*p* = 0.085), FA (*p* = 0.10), Stroop Color (*p* = 0.10), Interference (*p* = 0.10), Verbal Fluency (*p* = 0.10); myristic acid (C14:0), which had negative correlation with verbal fluency (*p* = 0.056) and trended negatively with IND (*p* = 0.07), Interference (*p* = 0.06), and SDMT (*p* = 0.085); and lauric acid (C12:0), which negatively correlated with TFC (*p* = 0.05), FA (*p* = 0.044), IND (*p* = 0.034), Verbal Fluency (*p* = 0.05), positively with TMS (*p* = 0.05), and trended negatively with Stroop Color (*p* = 0.10) and Stroop Word (*p* = 0.08). As a class, DAG negatively correlated with IND scores (*p* = 0.04) and nearly all cognitive assessments, missing significance only on SDMT (*p* = 0.069). DAG (14:1/16:0) had a significant negative correlation with IND (*p* = 0.05), Stroop Color (*p* = 0.036), with a negative trend correlation with the rest of the cognitive assessments. DAG (16:0/16:0) increased with clinical progression for 8 of the 10 measured outcomes. Increasing circulating levels of 8 TAGs in the CSF also correlated with clinical progression, all of which had only saturated fatty acids (Fig. 2; Supplemental Table 2). This contrasts with circulating levels of TAGs and DAGs in plasma, which decreased with clinical progression. Of all PCs measured, only 2 had circulating CSF concentrations that increased with clinical progression across several outcomes: PC (16:0/16:0) (significance in 9 of 10 outcomes) and PC (16:0/18:1) (5 of 10 outcomes). Conversely, plasma concentrations of these two lipids decreased with clinical progression: PC (16:0/16:0) had a significant correlation (2) or trend (3) for 5 of 10 outcomes, and PC (16:0/18:1) showed significant correlation (5) or trend (3) for 8 of the 10 outcomes. No individual or total ceramide was found to correlate with any of the clinical outcomes in CSF (data not shown). Four SMs correlated with multiple clinical outcomes (Fig. 2; Supplemental Table 2). SM (18:0) levels correlated with TMS (*p* = 0.01), FA (*p* = 0.0011), IND (*p* = 0.0091), Stroop Color (p-0.0165), Stroop Word (*p* = 0.0027), Stroop Interference (*p* = 0.0323), Verbal Fluency (*p* = 0.0322) and SDMT (*p* = 0.0414). Higher levels of SM (14:0), SM (16:0) and SM (18:1) correlated with severity for TMS (*p* = 0.0383; 0.0532, 0.0388, respectively); FA (*p* = 0.0071; 0.0017,0.0025); IND (*p* = 0.0260; 0.0091,0.0167) and Stroop Word (*p* = 0.0061; 0.0316 and 0.0189), with SM (14:0) also having a significant correlation with Stroop Color (*p* = 0.0194).

## Discussion

To our knowledge, this pilot study is the first report in HD correlating metabolomic changes in plasma and CSF with clinical progression across both early (HD1 and 2) and more advanced stages (HD3 and 4) of the disease. Metabolomic analyses of both plasma and CSF were performed across a spectrum of disease severities to generate a broad exploratory profile for dynamic biological changes in HD.

**Figure 3:**
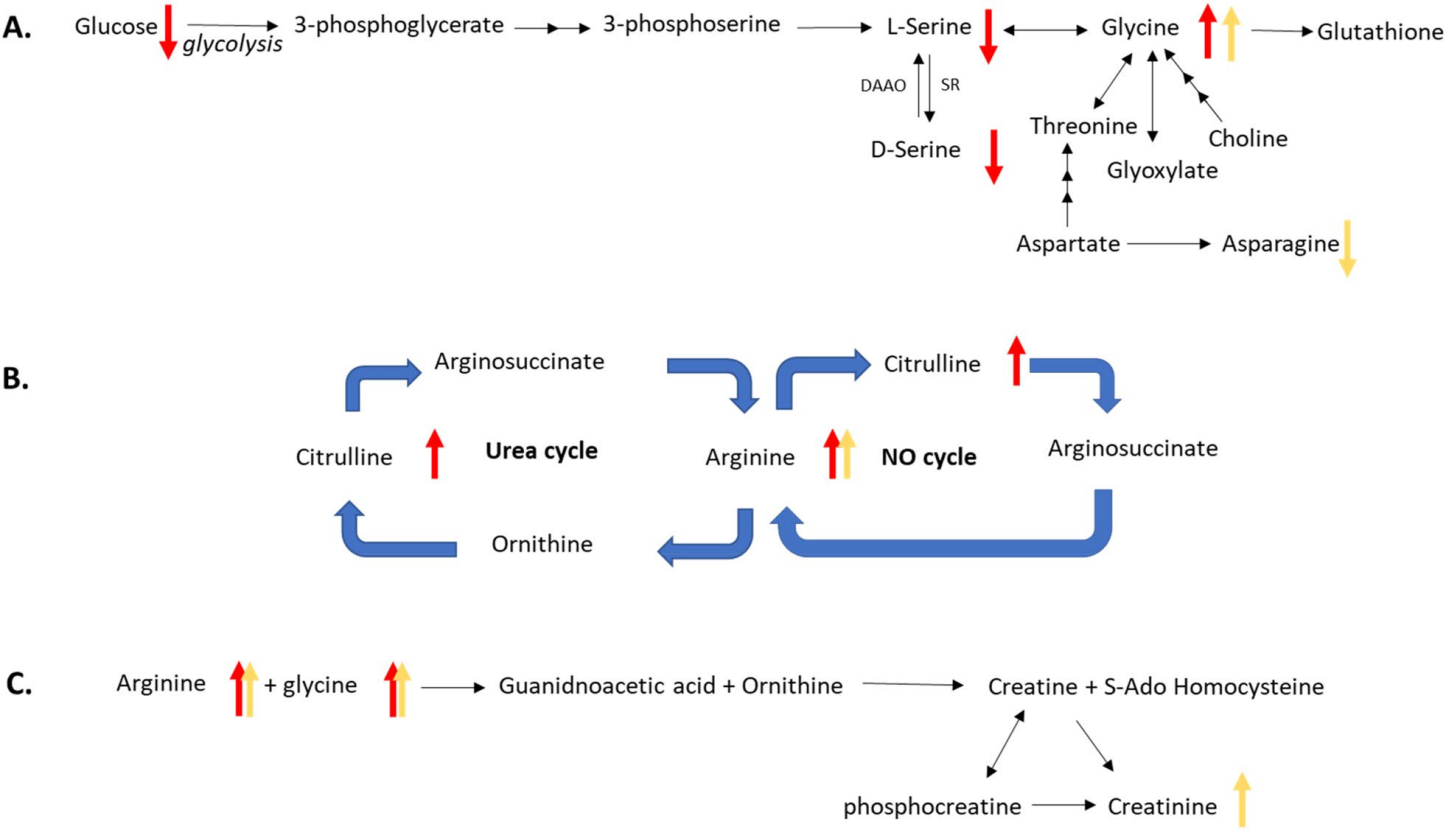
Overview of glycine (**A**), arginine (**B**), and creatine (**C**) metabolism in HD. Pathways with multiple metabolic changes are shown, including the urea cycle, nitrous oxide cycle and creatine synthesis. Arrows are red (plasma) or yellow (CSF), and indicate correlation with clinical state: up indicates circulating concentrations increase with progression, down indicates decrease with progression.

Here we report circulating levels of NAD^+^ in the CSF of HD participants for the first time. NAD^+^ is an important cofactor for several biochemical pathways, including glycolysis and three major classes of enzymes: SIRTuins, poly(ADP-ribose) polymerases (PARPs), and CD38/157 ecto enzymes^33,34^. NAD^+^ cellular levels regulate several pathways involved in mHtt toxicity^35^, including SIRT1 modulation of PGC-1α, SIRT-3 and PARP-1. SIRTuins and PARPs have been recently targeted for HD therapy^36,37^. Recent reports suggest neuroprotective effects for NAD^+^ in HD models, and suggest administration of nicotinamide riboside (NR), an NAD^+^ precursor, as a potential therapy^36^. Contrary to recent studies suggesting decreased levels of NAD^+^ would be expected with progressive neurodegeneration^38^, higher circulating CSF levels of NAD^+^ correlated to worsening clinical status in our study. The reason for this is not clear. Considerations may include decreased levels or function of CD38, a highly expressed glycoprotein in neurons and astrocytes that generates cyclic ADP-Ribose (cADPR) from NAD^+^ molecules^39^, or a proportional decrease in the intracellular concentration of NAD^+^. The latter case would be consistent with previous reports that NAD^+^ is preferentially released from intracellular stores in conditions of cell stress or inflammation^40^.Decreased intracellular levels of NAD^+^ are consistent with glutamate-induced excitotoxicity^41^, a process implicated in HD pathophysiology^42,43^. In the current study, levels of CSF glutamate were not determined, and increasing levels of plasma glutamate correlated only with declining behavioral scores. However, multiple pathways that indirectly modulate glutamatergic neurotransmission have also been proposed for involvement in neurodegenerative diseases, including signaling through the *N*-Methyl-D-aspartate (NMDA) receptor^44,45^. In this context, d-serine, a co-agonist for the NMDA receptor at the glycine site, has been investigated in neurodegeneration^43^. d-Serine plays a significant regulatory role in glutamate signaling, dendritic development, synaptic plasticity, long-term potentiation (LTP) and depression (LTD), and neuronal migration^44^. In this study, total plasma serine concentrations (l-serine and d-serine) positively correlated with two cognitive outcomes, while d-serine plasma concentrations correlated to one (Fig. 1), suggesting that both decreasing total serine and d-serine levels may be associated with clinical progression (Fig. 3). The total serine finding is consistent with previous studies showing decreased circulating plasma serine levels in HD relative to controls^45^. l-Serine is synthesized endogenously primarily from glucose via the glycolytic pathway (Fig. 3), while d-serine is synthesized from l-serine via serine racemase^44^. In our study, fasting glucose levels decreased with clinical progression, consistent with known impairment of glycolysis in HD and potentially influencing the low levels of total serine observed^46^. In CSF, no correlation was found between circulating levels of total serine or d-serine and clinical progression. We observed substantial inter-participant variation in d-serine CSF levels, which ranged from 0.61 to2.89 µM with a mean of 1.34 µM (Supplementary Table 4), consistent with previous reported values in healthy controls^47^.

Circulating plasma levels of glycine increased with clinical progression, consistent with previous studies^21,48,49^. No correlations were found in the CSF, largely due to levels at or below the limit of quantitation. However, circulating CSF glycine concentrations were higher for participants in later stages, similar to the clinical correlations in plasma. Elevated glycine with worsening clinical disease may be consistent with progressive excitotoxicity, a mechanism with numerous lines of evidence in HD^18^. The dichotomy between increasing glycine and decreasing total and d-serine levels in our study suggests either decreased metabolism of glycine or an increase in another glycine synthetic pathway. Glycine is mainly derived through de novo synthesis from the glycolytic pathway via serine, but may also derive from choline, threonine and glyoxylate (Fig. 3). Of these, only threonine was measured, which showed no association with progression. Choline, which is derived from PC in cell membranes, may increase via membrane breakdown from neurodegeneration causing release of membrane phospholipids^24,50^. In our study, an association between decreasing circulating plasma levels of PCs and clinical progression was observed. Future studies should determine circulating levels of choline and glyoxylate in HD subjects to further clarify these relationships. Upregulated plasma glycine may also indicate compensatory production of creatine and heme (Fig. 3), molecules derived from glycine that are involved in muscle/neuronal metabolism and proteins of the electron transport system, respectively^25^. Plasma creatinine showed no correlation to progression, whereas CSF creatinine significantly increased with worsening disease. It is not clear if this represents a preferential shift in CNS creatinine production and/or clearance^51^ as a response to failing energetic mechanisms. Of note, creatinine supplementation in the long-term CREST-E clincial trial did not influence functional progression^52^.

Nitric Oxide (NO) involvement in excitotoxicity has been well documented^53–55^. Increased formation of NO via nitric oxide synthase (NOS) depends upon an adequate supply of arginine (Fig. 3). Previous studies of increased activity for arginine synthase and arginine lyase suggests a role for the citrulline-NO cycle enzymes in excitotoxicity^55^. l-arginine is converted to L-citrulline and NO via NOS and the cofactor nicotinamide adenine dinucleotide phosphate (NADPH). Neuronal NOS mRNA is known to be reduced in HD striatum, particularly the dorsal caudate, and proportional to progression of disease^56^; this intraneuronal reduction, if present, may account for the accumulation of arginine with disease progression. The present data suggest that in both plasma and CSF higher circulating concentrations of arginine correspond to disease progression. These findings are consistent with previous reports^22,57^. Notably, the administration of l-arginine to HD transgenic mice accelerated motor phenotype and weight loss, while diets absent in l-arginine slowed weight loss^58^. Plasma citrulline also negatively correlated with cognitive outcomes in our data. Higher circulating concentrations of citrulline have been reported in HD patients relative to controls^23^. This may reflect lesser activity of arginosuccinate synthase or lyase, a consequence demonstrated in murine models of mutant huntingtin suppressing C/EBP alpha, a critical regulator of urea cycle enzymes resulting in elevated citrulline and hyperammonemia^23^. Protein restriction in these animals normalized urea cycle function, lessened mHtt aggregation, and improved phenotype. Skene and colleagues have recently reported significantly elevated arginine and citrulline in transgenic HD sheep^59^. Taken together, additional investigation of the urea cycle in HD is warranted.

While no relation was observed in plasma between fatty acids and clinical outcomes, increasing circulating CSF levels of several saturated long chain fatty acids (LCFAs) correlated with disease progression, including stearic acid, margaric acid, palmitic acid, myristic acid and lauric acid. De novo fatty acid synthesis is closely associated with inflammatory cell subsets^60^ and hypothesized to drive inflammation and disease activity for neurological conditions including Huntington’s Disease^60^. Of note, fatty acid dysregulation in HD has been previously identified, with suggestion that brain fatty acids might be regulated differently and independently of circulating fatty acid concentrations^24^. Other reports indicate no difference in levels from controls or premanifest individuals compared to symptomatic persons^61^. Our results are consistent with recent data showing striatal astrocytes in the HdhQ (150/150) mouse model switch from glycolysis to fatty acid oxidation in the setting of decreasing glucose levels, a process involving increasing fatty acid concentrations that temporarily stabilize energetics but at the eventual expense of producing oxidative damage^62^. It may be that our divergent fatty acid results between plasma and CSF reflect an emerging preference in the brain, with progression, for high-energy substrates at the site of greatest vulnerability. Furthermore, our data show circulating plasma levels are not representative of circulating CSF levels, and therefore do not appear useful as prognostic lipid biomarkers.

Fatty acids are precursors for DAGs, TAGs, and phospholipid synthesis regulated by sterol regulatory element response protein 2 (SREBP-2), an enzyme known to be downregulated in murine HD models and manifest human postmortem brain^24^. Conversely, lipoprotein lipase (LPL)-mediated hydrolysis of triacylglycerol is an important source of diacylglycerols, free fatty acids and phospholipids in the brain^25^ and is thought to influence responses to oxidative stress in several neurodegenerative disorders^63^. Therefore, the concomitant increase of circulating saturated LCFAs in CSF with corresponding DAGs, TAGs, phospholipids and sphingolipids is perhaps not surprising. In our study, increasing circulating CSF levels of DAG (12:0/18:1),DAG (14:0/14:0),DAG (14:0/16:0),DAG (14:1/16:0), DAG (16:0/16:0), and DAG(16:0/18:0) were observed, with DAG (16:0/16:0) having the strongest association with progression. Whereas eicosanoic acid did not correlate with any measured outcome, DAG (20:0/20:0) increased with clinical progression across 7 of the 10 measured outcomes. Similarly, increasing circulating CSF levels of 8 identified TAGs correlated with progression, all of which incorporated only saturated LCFAs. TAG increases in the CSF may reflect progressive compensatory efforts in energy-depleted environments. Whether DAG accumulation reflects a reaction to energetic abnormalities with disease progression (perhaps derived from TAG) or more specific second- messenger signaling activity is unclear; DAG is a highly active second messenger across numerous signaling cascades (the IP3-DAG system)^30^. Notably, none of the increasing DAGs or TAGs with clinical correlations in the CSF were found to have significant correlations in plasma; moreover, in contrast to the CSF, decreasing circulating plasma levels of the eight DAGs and 92 TAGs correlated with clinical progression. Decreasing levels of TAGs in plasma are consistent with decreasing circulating plasma glucose levels, as TAG synthesis occurs via the glycerol-3-phosphate pathway in endoplasmic reticulum and mitochondria^64^. Taken together, our data suggest substantially different metabolism for TAG and DAG species between peripheral tissues and brain in HD, an observation for which therapeutic implications are not yet clear.

In the CSF, increasing circulating levels of two PCs, both of which incorporated palmitic acid (PC (16:0/16:0) and PC (16:0/18:1)), correlated with clinical progression. These are two of the four most abundant PCs in human CSF, with PC (16:0/18:1) being the most abundant^65^. Of all PCs and DAGs assayed, PC (16:0/16:0) and DAG (16:0/16:0) associated most strongly with progression, correlating with 9/10 and 8/10 measured outcomes, respectively. The concomitant increase of circulating CSF levels of palmitic acid, DAG(16:0/16:0) and PC (16:0/16:0) would suggest an increase in SM (16:0) based on the sphingomyelin cycle, in which sphingomyelin synthase transfers a phosphorylcholine group from a PC to ceramide to generate DAG and SM^66^. Indeed, we observed SM (16:0) increasing with clinical progression, whereas increasing sphingomyelins 14:0, 18:0, and 18:1 also appeared to have a strong relationship with clinical decline. Increasing levels may simply reflect progressive neurodegeneration and membrane breakdown. However, a defective de novo biosynthetic pathway for sphingolipids has been demonstrated in multiple HD pre-clinical models. For example, decreases in dihydrosphingosine, dihydrosphingosine-1-phosphate and dihydroceramide 18:0 were observed in R6/2 mice^67^. Of note, no concomitant change in circulating ceramide levels was observed with progression in our data. This may reflect other peripheral sources of ceramide, as it is known to readily cross the blood–brain barrier, or possibly a preferential compensatory preservation of CSF levels^68^. Ceramide is degraded to sphingosine and phosphorylated to form sphingosine-1-phosphate (S1P), a molecule which may have relevance in HD^67,69^. There is suggestion that sphingosine-1-phosphate (S1P) species may be especially important in HD compensatory mechanisms, supporting glial survival, neurite outgrowth, blood–brain barrier integrity, and neurogenesis^67^.

Several limitations to these pilot data should be acknowledged. Our findings must be interpreted with caution in light of the small sample size (n = 12) and unadjusted statistical analysis for multiple comparisons. Highlighted results obtained are trends or carry nominal significance, which are limited by the small sample and potential variability in studying both early and advanced stages in HD. An additional limitation is the absence of an age-matched healthy control group. These factors limit our ability to more specifically assess associations with disease progression. CSF was not quality checked for blood contamination, making contamination with blood metabolites possible, though on inspection of fluid and analysis of metabolite concentrations this risk was felt to be minimal. Behavioral and anti-chorea medications were allowed in the study, as washout of these treatments and the related risks for instability were not felt to be justified. As such, it is possible that clinical results were influenced by this decision. Of note, chorea is only one component of the Total Motor Score, which also assesses voluntary motor capability, gait, and other involuntary elements like bradykinesia, rigidity and dystonia. It is important to recognize CSF metabolite levels reflect the extracellular environment of the CNS and may not entirely or accurately reflect intracellular changes.

## Conclusion

Overall, these data are advantageous in providing a simultaneous look at both plasma and CSF metabolites, and allow for a novel cross-sectional analysis across a broad spectrum of the disease. A number of changes consistent with previously reported metabolites were observed in our data and help generate hypotheses for future work. In particular, urea cycle defects appear to deserve further study for potential therapeutic implications, as do d-serine, glycine, and NAD^+^. Larger longitudinal studies are planned to clarify the biological and potentially therapeutic relevance of these findings.

## Methods

Ethical approval for conduct of this study was obtained from the Institutional Review Board of Cooper University Hospital at Rowan University on July 18, 2017. All participants were over the age of 18, and all provided written informed consent to participate in the study. This pilot study was done in accordance with the Declaration of Helsinki and International Conference on Harmonization Good Clinical Practice guidelines. Twelve consenting participants with either genetically confirmed HD or an unequivocal phenotype (Diagnostic Confidence Level IV, UHDRS) and a genetically confirmed first-degree relative were enrolled from September 30, 2017 to July 18, 2018. Participants were stratified according to Total Functional Capacity Score^70^. This is a 13-point scale that assesses functional performance in 5 domains: capacity for work, finances, domestic chores, self-care, and care level required. Scores range from 13 (normal) to 0 (total incapacitation) and define stages of the disease: HD1 (TFC 11–13, early mild), HD2 (TFC 7–10, mild), HD3 (TFC 4–6, moderate, and HD4 (0–3, severe). All study visits were conducted in the morning at Cooper University Hospital (Camden, NJ). Participants were advised to arrive fasted the morning of study. Study visits consisted of vital signs, review of concomitant medications and medical conditions, performance of the Unified Huntington’s Disease Rating Scale (UHDRS) by a certified examiner (AM), blood draw, and fluoroscopically guided lumbar puncture. The Unified Huntington’s Disease Rating Scale is comprised of five domains for assessment: Motor, Cognitive, Behavioral, Independence Scale, and Functional Capacity^16^. Increasing scores in the Motor domain reflect worsening disease features, while decreasing scores in Cognitive, Behavioral, Independence, and Functional measurements reflect worsening disease. 20 cc of blood was withdrawn into sodium heparin tubes and centrifuged at 3400 RPM for 15 min, after which plasma was aliquoted and stored at − 80 °C. Lumbar punctures were performed by study personnel (AM, HS) and withdrew approx. 4 cc of clear cerebrospinal fluid per participant for storage at − 80 °C. QC for blood contamination of CSF was not performed.

Metabolites shown in “Results” section were selected for (1) having a minimum of 10 samples, (2) having nominally significant changes across motor, functional, and cognitive domains, or (3) for special interest as being nominally significant for motor change only or multiple cognitive domains only. Metabolite changes of nominal significance across multiple facets of the disease phenotype were felt to most likely reflect underlying biology. Behavior significance was not required for meeting this threshold, given the allowance of behavioral treatment during the study (participants on anxiolytics or antidepressants). Tetrabenazine treatment for chorea was also allowed in the study, as chorea is only one component of Total Motor Score assessment.

### Metabolomics panels

Metabolites were extracted from plasma and cerebrospinal fluid (CSF) and concentrations obtained using the AbsoluteIDQ kit p180 (Biocrates Life Science AG, Austria) following the manufacturer’s protocol for the API5500 LC/MS/MS System (ABSciex, USA), running with Analyst 1.5.2 software equipped with an electrospray ionization source, a Shimadzu CBM-20A command module, LC-20AD pump, and a Shimadzu SIL-20AC-HT autosampler and a CTO-10Ac column oven heater^71^.

The Lipidyzer (Sciex, Framingham, MA) platform was used to measure lipids in the phosphatidylcholine (PC), phosphatidylethanolamine (PE), lysophosphatidylcholine (LPC), lysophosphatidylethanolamine (LPE), free fatty acid (FFA), sphingomyelin (SM), diacylglycerol (DAG), cholesteryl ester (CE), ceramide (CER), hexosylceramide (HCER), lactosylceramide (LCER), dihydroceramide (DCER), and triacylglycerol (TAG) groups of lipids on API5500 LC/MS/MS System (ABSciex, USA), following a previously describe method^72^. Lipid species were included in the data analyses if above the limit of quantification in > 90% of the participants was satisfied.

### Kynurenine pathway

Separation of the kynurenines was accomplished following a previously published protocol^73^. Briefly, to 40 µl plasma or CSF, 10 µl internal standard and 10 µl 0.1% formic acid in water was added. Solid-phase extraction cartridges (Oasis HLB, Waters Corp) were conditioned with 1 ml methanol, then 1 ml water. The samples were added and washed with 100 µl water. Finally, the metabolites were eluted with 1 ml 0.1% formic acid in 95:5 methanol: water and stream-dried under nitrogen. The samples were reconstituted in 100 µl 0.1% formic acid in 10:90 methanol: water and transferred to auto sampler vials for analysis. Data was acquired using a Nexera XR HPLC (Shimadzu) coupled with a QTRAP 6500 + (SCIEX) and was analyzed with Analyst 1.6 (SCIEX). A linear gradient was run for 30 min at a flow rate of 0.3 ml/min: 0–1 min 5% B, 3 min 23% B, 3.1–5 min 70% B, 5.5–20 min 90% B, 20.1 min 10% B, 21 min 5% B at 40 °C, on an X-Select HSS C18 column (2.1 × 150 mm, 2.5 µm, Waters), with mobile phase A consisting of 0.2% aqueous formic acid and mobile phase B consisting of 0.2% formic acid in methanol. Relative concentrations (abundance) of the metabolites were determined in standard solution and not matrix (matrix effects were not considered) using area ratios calculated using their corresponding deuterated standard with the exception of 3-hydroxykynurenine, and anthranilic acid, where D_4_-Kyn was used as their internal standard.

### d-Serine

d-Serine levels were measured following a previously developed protocol with slight modifications^74^. Briefly, plasma or CSF samples (100 μl) were combined with 20 μl aliquot of IS (10 nmol/ml in acetone) and 400 μl acetone and then centrifuged at 13,000×*g* for 10 min at 4 °C. A 400 μl aliquot of the supernatant was subsequently derivatized with 300 μl (R)-1-Boc-2-piperidinecarbonyl chloride. After derivatization by stirring at 1000 rpm for 2 h at room temperature, 200 μl of triflouroacetic acid was added to each sample and then incubated for 1 h. The terminal product was evaporated to dryness under a stream of nitrogen. The residue was dissolved in 100 μl of methanol/water, 10:90, v/v and transferred to the autosampler for analysis.

### NAD^+^ metabolome

Separation of the NAD^+^ metabolites was accomplished using a previously described method^32^. Briefly, 20 μl of CSF was solubilized in 60 μl of methanol, including the addition of 5 μl of internal standard. Samples were then centrifuged at 4 °C for 10 min at 13,200×*g* to remove the protein pellet. The supernatant was collected and placed in an autosampler vial for analysis. Three quality controls with spiked standards was used for determination of the relative concentrations of NAD^+^ and its metabolites. The NAD^+^ metabolites were resolved using an Accucore HILIC column (2.1 × 150 mm, 2.6 μ, Thermo) at 32 °C was used with ammonium acetate [7.5 mM, pH 7.86] as mobile phase A and acetonitrile as mobile phase B with a 10 µl injection volume. The following linear gradient was run for 14.0 min at a flow rate of 0.4 ml/min: 0–1 min 90% B, 1.5 min 72.5% B, 2.5 min 67.5% B, 8.0 min 20% B, 10 min 20% B, 10.1 min 90% B. Relative values for the metabolites were determined using area ratios of the targeted metabolites and the corresponding internal standard using the following heavy standards: C_13_-NAD, C_13_-NADH, C_13_-ADPR, C_13_-AcCoA. Calibration curves were carried out in standard solutions. Matrix effects were accounted for in each targeted matrix by adjusting calculated levels based on three quality controls (low, middle, high).

### Statistical analysis

Pearson correlation coefficients were calculated between the components of the UHDRS as continuous variables and the measured metabolites. P-values obtained were not adjusted for multiple comparisons, and so all values are considered nominally significant. Correlations were categorized as moderate (absolute r-value = 0.4–0.69) or strong (absolute r value = 0.7 to 1.0), and coded accordingly in heat maps (moderate as lighter hue, strong as darker hue). Correlations could be positive (metabolite concentrations changing in same direction as clinical scale) or negative (concentrations changing in opposite direction as clinical scale). Among domains of the UHDRS, TMS and Behavior scores tend to increase with disease progression, while TFC, IS, FA, and cognitive assessments tend to decrease with progression; therefore, with worsening disease, correlations for TMS and Behavior were expected to be of opposite polarity to other assessments. Considerations of normality and confounding variables were not considered, as this was a pilot study with only 12 subjects.

## Supplementary information

Supplementary Information.
