# Author Correction: Cross-sectional analysis of plasma and CSF metabolomic markers in Huntington’s disease for participants of varying functional disability: a pilot study

**DOI:** 10.1038/s41598-021-89167-7

**Published:** 2021-05-05

**Authors:** Andrew McGarry, John Gaughan, Cory Hackmyer, Jacqueline Lovett, Mohammed Khadeer, Hamza Shaikh, Basant Pradhan, Thomas N. Ferraro, Irving W. Wainer, Ruin Moaddel

**Affiliations:** 1 Department of Neurology, Cooper University Hospital and Cooper Medical School of Rowan University, Camden, NJ USA; 2 Biomedical Research Center, National Institute On Aging, National Institutes of Health, Baltimore, MD 21224 USA; 3 Department of Biomedical Sciences, Cooper Medical School of Rowan University, Camden, NJ USA

Correction to: *Scientific Reports* 10.1038/s41598-020-77526-9, published online 24 November 2020

This Article contains errors in the last sentence from the Introduction section.

"This was carried out using several metabolomic platforms (Biocrates p180 kit, ABSciex Lipidyzer platform, LC-MS/MS method for NAD^+^ and kynurenine) in both plasma and the corresponding CSF from participants with HD ranging from TFC Stage I (mild) to IV (severe)."

should read:

"This was carried out using several metabolomic platforms (Biocrates p180 kit, ABSciex Lipidyzer plat-form, LC-MS/MS method for NAD^+^ and kynurenine) in both plasma and the corresponding CSF from participants with HD ranging from TFC Stage I (mild) to III (moderate-severe)."

Secondly, in the first paragraph of the Results section,

"Baseline TFC ranged from 3 to 13; three participants were HD Stage I (scores 12, 13, 13), four were Stage II (7, 8, 8, 9), four Stage III (4, 5, 6, 6), and one Stage IV (3)."

should read:

"Baseline TFC ranged from 3 to 13; three participants were HD Stage I (scores 12, 13, 13), four were Stage II (7, 8, 8, 9), and five Stage III (3, 4, 5, 6, 6)."

Furthermore, in the first sentence of the Discussion section,

“To our knowledge, this pilot study is the first report in HD correlating metabolomic changes in plasma and CSF with clinical progression across both early (HD1 and 2) and more advanced stages (HD3 and 4) of the disease.”

should read:

“To our knowledge, this pilot study is the first report in HD correlating metabolomic changes in plasma and CSF with clinical progression across both early (HD1 and 2) and more advanced stages (HD3) of the disease.”

Lastly, in the Methods section,

“Scores range from 13 (normal) to 0 (total incapacitation) and define stages of the disease: HD1 (TFC 11–13, early mild), HD2 (TFC 7–10, mild), HD3 (TFC 4–6, moderate, and HD4 (0–3, severe).”

should read:

“Scores range from 13 (normal) to 0 (total incapacitation) and define stages of the disease: HD1 (TFC 11-13), HD2 (TFC 7-10), HD3 (TFC 3-6), HD4 (1-2), and HD5 (0)."

