# Supplementary material for: Cross-sectional analysis of plasma and CSF metabolomic markers in Huntington’s disease for participants of varying functional disability: a pilot study
Source: Sci Rep. 2020 Nov 24;10:20490. doi: 10.1038/s41598-020-77526-9 (PMC7686309; doi:10.1038/s41598-020-77526-9)
Supplement: Supplementary file 1 — Supplementary Information. [file 41598_2020_77526_MOESM1_ESM.docx]

**Cross-Sectional Analysis of Plasma and CSF Metabolomic Markers in Huntington’s Disease for Participants of Varying Functional Disability: A Pilot Study**

Andrew McGarry^1a^, John Gaughan^1^, Cory Hackmyer^1^, Jacqueline Lovett^2^, Mohammed Khadeer^2^, Hamza Shaikh^1^, Basant Pradhan^1^, Thomas N. Ferraro^3^, Irving W. Wainer^1^, Ruin Moaddel^2b^

1. Department of Neurology, Cooper University Hospital and Cooper Medical School of Rowan University, Camden, NJ, USA

2. Biomedical Research Center, National Institute on Aging, National Institutes of Health, Baltimore, Maryland, 21224, USA.^1^Chronobiology, Faculty of Health and Medical Sciences, University of Surrey, Guildford GU2 7XH, United Kingdom

3. Department of Biomedical Sciences, Cooper Medical School of Rowan University

^2^Preclinical, Imaging & Research Laboratories (PIRL), SAHMRI, Gilles Plains, Adelaide, Australia

^3^National Institute for Public Health and the Environment (RIVM), 3720 BA Bilthoven, The Netherlands

^4^South Australian Research and Development Institute, Roseworthy, South Australia

^5^Department of Physiology, Development and Neuroscience, University of Cambridge, Downing Street, Cambridge CB2 3DY, United Kingdom

^1^Chronobiology, Faculty of Health and Medical Sciences, University of Surrey, Guildford GU2 7XH, United Kingdom

^2^Preclinical, Imaging & Research Laboratories (PIRL), SAHMRI, Gilles Plains, Adelaide, Australia

^3^National Institute for Public Health and the Environment (RIVM), 3720 BA Bilthoven, The Netherlands

^4^South Australian Research and Development Institute, Roseworthy, South Australia

^5^Department of Physiology, Development and Neuroscience, University of Cambridge, Downing Street, Cambridge CB2 3DY, United Kingdom

^a^Email:McGarry-Andrew@CooperHealth.edu

^b^Email: Moaddelru@grc.nia.nih.gov

**Correspondence and requests for materials should be addressed to A.M. (email: McGarry-Andrew@CooperHealth.edu) or R.M. (email: Moaddelru@grc.nia.nih.gov)**

**Supplemental Table 1.** Metabolites with nominally significant r-values in plasma, ranging from -1 to 1. P-values are listed beneath r-values. p-values are listed beneath r-values. p-values < 0.05 are in Bold. (N) represents the number of participants for whom circulating metabolite levels were determined.

|  | **TFC** | **TMS** | **FA** | **INDEP** | **SCOLOR** | **SWORD** | **SINTERF** | **VERBAL** | **SDMT** | **BEHAV** |
| --- | --- | --- | --- | --- | --- | --- | --- | --- | --- | --- |
| **Arg (12)** | -0.47504 0.1186 | **0.58985 0.0435** | -0.41616 0.1784 | -0.51490 0.0867 | **-0.57966 0.0482** | **-0.60748 0.0362** | **-0.66072 0.0193** | **-0.67094 0.0169** | **-0.69506 0.0121** | -0.20892 0.5146 |
| **Cit (12)** | -0.42680 0.1665 | 0.41859 0.1756 | -0.51847 0.0842 | **-0.59802 0.0400** | **-0.60578 0.0368** | -0.49965 0.0981 | **-0.62725 0.0290** | -0.56278 0.0568 | -0.49459 0.1021 | 0.11282 0.7270 |
| **Gly (12)** | **-0.61371 0.0338** | **0.60039 0.0390** | -0.46985 0.1233 | **-0.60290 0.0380** | -0.56904 0.0535 | -0.35660 0.2552 | -0.57055 0.0527 | -0.56327 0.0565 | -0.51465 0.0869 | -0.19344 0.5469 |
| **Val (12)** | 0.48445 0.1105 | -0.56935 0.0533 | 0.34789 0.2678 | 0.50573 0.0935 | 0.35411 0.2588 | 0.25240 0.4287 | 0.41301 0.1821 | 0.46307 0.1295 | 0.39333 0.2059 | **0.59982 0.0392** |
| **Ser (12)** | 0.34251 0.2758 | -0.27981 0.3784 | 0.41095 0.1845 | 0.45372 0.1385 | **0.65330 0.0212** | 0.44637 0.1458 | **0.60489 0.0372** | 0.43620 0.1563 | 0.40054 0.1970 | -0.37568 0.2288 |
| **DSER (12)** | 0.35013 0.2645 | -0.33840 0.2820 | 0.34696 0.2692 | 0.51302 0.0881 | 0.47532 0.1184 | **0.58130 0.0474** | 0.41151 0.1838 | 0.48601 0.1092 | 0.52273 0.0812 | -0.43732 0.1551 |
| **KYNUR (12)** | 0.48964 0.1061 | -0.56763 0.0542 | 0.32094 0.3091 | 0.41801 0.1763 | 0.20810 0.5163 | 0.25373 0.4262 | 0.34090 0.2782 | 0.43958 0.1528 | 0.38762 0.2131 | **0.80533 0.0016** |
| **H1 (12)** | 0.53508 0.0730 | -0.54385 0.0676 | 0.39337 0.2058 | 0.52495 0.0797 | 0.25130 0.4308 | 0.31187 0.3237 | 0.40771 0.1883 | 0.50143 0.0967 | 0.45458 0.1376 | **0.60405 0.0375** |
| **SM 20:0 (12)** | **0.79634 0.0019** | **-0.72681 0.0074** | **0.79651 0.0019** | **0.65123 0.0218** | **0.63378 0.0269** | **0.62402 0.0301** | **0.63937 0.0252** | **0.62958 0.0283** | **0.57952 0.0483** | 0.09407 0.7712 |
| **SM 20:1 (12)** | **0.73931 0.0060** | **-0.63466 0.0266** | **0.70120 0.0111** | 0.53835 0.0710 | 0.56417 0.0560 | 0.48190 0.1126 | **0.59008 0.0434** | 0.54068 0.0695 | 0.48660 0.1087 | 0.14645 0.6497 |
| **SM 22:0 (12)** | **0.64229 0.0243** | **-0.66233 0.0189** | **0.76471 0.0038** | 0.57603 0.0500 | 0.40713 0.1890 | **0.62933 0.0283** | 0.40442 0.1923 | 0.52217 0.0816 | 0.47685 0.1170 | 0.09621 0.7661 |
| **SM 22:1 (12)** | **0.58257 0.0468** | **-0.65251 0.0215** | **0.62760 0.0289** | 0.48762 0.1078 | 0.35377 0.2593 | 0.37265 0.2329 | 0.41407 0.1808 | 0.42873 0.1643 | 0.38896 0.2114 | 0.56602 0.0551 |
| **SM 24:0 (12)** | **0.60663 0.0365** | **-0.60063 0.0389** | **0.76377 0.0038** | **0.68134 0.0147** | **0.57673 0.0496** | 0.52122 0.0823 | 0.54421 0.0674 | 0.49501 0.1018 | 0.47468 0.1189 | -0.00653 0.9839 |
| **CE (12)** | 0.53042 0.0760 | -0.53906 0.0705 | 0.49043 0.1055 | 0.48970 0.1061 | 0.53152 0.0753 | 0.72054 0.0082 | 0.44704 0.1451 | **0.58061 0.0478** | 0.57532 0.0503 | -0.50413 0.0947 |
| **DAG (12)** | 0.45312 0.1391 | **-0.64648 0.0231** | 0.56824 0.0539 | **0.62235 0.0307** | 0.39727 0.2010 | 0.46401 0.1286 | 0.49921 0.0985 | 0.50135 0.0968 | 0.49818 0.0993 | **0.69817 0.0116** |
| **PC (12)** | **0.80531 0.0016** | **-0.75593 0.0045** | **0.72036 0.0082** | **0.67290 0.0165** | **0.65951 0.0196** | **0.74724 0.0052** | **0.69280 0.0125** | **0.72146 0.0081** | **0.71216 0.0094** | -0.10942 0.7350 |
| **PE (12)** | **0.75298 0.0047** | **-0.76338 0.0039** | **0.58384 0.0462** | **0.66378 0.0186** | **0.73416 0.0066** | **0.67148 0.0168** | **0.82564 0.0009** | **0.74527 0.0054** | **0.80610 0.0015** | 0.10357 0.7487 12 |
| **SM (12)** | **0.67245 0.0166** | **-0.63249 0.0273** | **0.67468 0.0161** | 0.53098 0.0757 | 0.49800 0.0994 | **0.65175 0.0217** | 0.47475 0.1189 | 0.55038 0.0637 | 0.53612 0.0724 | -0.16596 0.6062 |
| **TAG (12)** | 0.44454 0.1476 | **-0.62056 0.0313** | 0.56440 0.0559 | 0.60854 0.0357 | 0.36220 0.2473 | 0.43260 0.1601 | 0.46514 0.1276 | 0.48064 0.1137 | 0.46268 0.1299 | **0.70832 0.0099** |
| **CE 16:0 (12)** | 0.57305 0.0515 | **-0.59727 0.0403** | 0.47598 0.1178 | 0.54157 0.0690 | **0.59965 0.0393** | **0.76354 0.0039** | 0.55991 0.0583 | **0.65816 0.0200** | **0.68100 0.0148** | -0.46765 0.1253 |
| **CE 20:1 (10)** | 0.46894 0.1716 | -0.47754 0.1628 | 0.24221 0.5002 | 0.53638 0.1100 | **0.67641 0.0317** | **0.67379 0.0327** | **0.72979 0.0166** | **0.78637 0.0070** | **0.86435 0.0013** | -0.50781 0.1340 |
| **DAG 16:0/18:1 (12)** | 0.47467 0.1189 | **-0.62069 0.0313** | 0.52207 0.0817 | **0.59622 0.0407** | 0.34644 0.2700 | 0.41915 0.1750 | 0.46800 0.1249 | 0.48301 0.1117 | 0.48443 0.1105 | **0.69080 0.0129** |
| **DAG 16:1/18:1 (12)** | **0.61340 0.0339** | **-0.65773 0.0201** | 0.46255 0.1300 | **0.60534 0.0370** | 0.41073 0.1847 | 0.36007 0.2503 | 0.52893 0.0770 | 0.56626 0.0549 | 0.53099 0.0757 | **0.79269 0.0021** |
| **DAG 16:1/18:2 (12)** | 0.53939 0.0703 | **-0.64282 0.0242** | 0.50425 0.0946 | **0.60390 0.0376** | 0.39391 0.2052 | 0.36988 0.2366 | 0.51360 0.0876 | 0.53463 0.0733 | 0.49558 0.1013 | **0.80950 0.0014** |
| **DAG 18:0/18:1 (12)** | 0.45022 0.1419 | **-0.61094 0.0348** | 0.52446 0.0800 | 0.57516 0.0504 | 0.30400 0.3367 | 0.43375 0.1589 | 0.41339 0.1816 | 0.46741 0.1255 | 0.45867 0.1337 | **0.68254 0.0145** |
| **DAG 18:0/18:2 (11)** | 0.44377 0.1715 | **-0.61085 0.0459** | **0.61154 0.0456** | 0.55499 0.0764 | 0.30413 0.3632 | 0.45234 0.1624 | 0.41530 0.2040 | 0.42854 0.1885 | 0.41284 0.2070 | **0.65076 0.0301** |
| **DAG 18:1/18:1 (12)** | **0.54630 0.0661** | **-0.76597 0.0037** | **0.63880 0.0254** | **0.71114 0.0095** | 0.53743 0.0715 | **0.61466 0.0334** | **0.61755 0.0324** | **0.63716 0.0259** | **0.62862 0.0286** | **0.67692 0.0156** |
| **DAG 18:1/18:2 (12)** | 0.35996 0.2504 | **-0.63214 0.0274** | **0.60898 0.0356** | **0.61770 0.0323** | 0.46644 0.1264 | 0.54508 0.0668 | 0.52830 0.0775 | 0.48972 0.1061 | 0.49702 0.1002 | 0.57241 0.0518 |
| **DAG 18:2/20:4 (10)** | 0.32011 0.3672 | **-0.65720 0.0389** | 0.55343 0.0970 | **0.66307 0.0366** | **0.63567 0.0482** | **0.70755 0.0221** | **0.63591 0.0481** | 0.56574 0.0883 | **0.66270 0.0368** | 0.19701 0.5854 |
| **PC 16:0/16:0 (12)** | **0.61497 0.0333** | -0.51745 0.0849 | 0.46589 0.1269 | 0.39217 0.2074 | 0.44604 0.1461 | 0.54756 0.0654 | 0.49635 0.1007 | 0.51276 0.0883 | 0.55878 0.0590 | -0.18338 0.5683 |
| **PC 16:0/18:0 (12)** | **0.62082 0.0312** | **-0.55113 0.0633** | 0.56862 0.0537 | 0.46611 0.1267 | 0.37869 0.2248 | 0.57642 0.0498 | 0.39950 0.1982 | 0.50742 0.0922 | 0.53212 0.0749 | -0.04827 0.8816 |
| **PC 16:0/18:1 (12)** | **0.72654 0.0074** | **-0.67048 0.0170** | 0.50142 0.0968 | 0.54298 0.0681 | 0.47779 0.1162 | **0.61639 0.0328** | 0.53122 0.0755 | **0.61877 0.0319** | **0.63371 0.0269** | 0.00543 0.9866 |
| **PC 16:0/18:2 (12)** | **0.78267 0.0026** | **-0.66165 0.0191** | **0.70262 0.0108** | **0.59563 0.0410** | **0.60711 0.0363** | **0.64956 0.0223** | **0.63690 0.0259** | **0.63425 0.0268** | **0.59941 0.0394** | -0.10824 0.7378 |
| **PC 16:0/20:1 (12)** | **0.79527 0.0020** | **-0.76797 0.0035** | **0.68308 0.0143** | **0.83540 0.0007** | **0.82319 0.0010** | **0.75985 0.0041** | **0.88302 0.0001** | **0.83481 0.0007** | **0.85253 0.0004** | -0.04666 0.8855 |
| **PC 16:0/20:2 (12)** | **0.65312 0.0213** | -0.56874 0.0536 | 0.45796 0.1344 | **0.61302 0.0340** | **0.65308 0.0213** | 0.32330 0.3053 | **0.71535 0.0089** | 0.56737 0.0544 | **0.60385 0.0376** | 0.25357 0.4265 |
| **PC 16:0/22:5 (12)** | **0.58944 0.0437** | **-0.58789 0.0444** | 0.42916 0.1639 | 0.51422 0.0872 | 0.48789 0.1076 | **0.66792 0.0176** | 0.47305 0.1204 | **0.63648 0.0261** | **0.65592 0.0206** | -0.32943 0.2957 |
| **PC 18:0/18:0 (12)** | **0.62643 0.0293** | **-0.62093 0.0312** | 0.51212 0.0887 | 0.54689 0.0658 | 0.35229 0.2614 | **0.59496 0.0413** | 0.40103 0.1964 | 0.56504 0.0556 | **0.57997 0.0481** | 0.03677 0.9097 |
| **PC 18:0/18:2 (12)** | **0.63426 0.0268** | **-0.58224 0.0470** | **0.67774 0.0154** | 0.50464 0.0943 | 0.36843 0.2386 | **0.61909 0.0318** | 0.39154 0.2082 | 0.50859 0.0913 | 0.46735 0.1255 | -0.07829 0.8089 |
| **PC 18:1/16:1 (12)** | **0.68114 0.0147** | **-0.71785 0.0086** | 0.39437 0.2046 | 0.46244 0.1301 | 0.51936 0.0835 | **0.59472 0.0414** | **0.58677 0.0449** | **0.66681 0.0179** | **0.67475 0.0161** | 0.30769 0.3306 |
| **PC 18:2/16:1 (12)** | **0.63928 0.0252** | **-0.62759 0.0289** | **0.58960 0.0436** | 0.47110 0.1221 | **0.63487 0.0266** | **0.61008 0.0352** | **0.60901 0.0356** | **0.58392 0.0462** | 0.54276 0.0682 | -0.03297 0.9190 |
| **PE 16:0/18:1 (12)** | **0.67549 0.0159** | **-0.66029 0.0194** | 0.55827 0.0592 | **0.70314 0.0107** | **0.65392 0.0211** | 0.44741 0.1447 | **0.76317 0.0039** | **0.63231 0.0274** | **0.65229 0.0215** | 0.49407 0.1025 |
| **PE 16:0/18:2 (12)** | **0.76030 0.0041** | **-0.75279 0.0047** | **0.63551 0.0264** | **0.73270 0.0067** | **0.62403 0.0301** | 0.49564 0.1013 | **0.74314 0.0056** | **0.67116 0.0169** | **0.65538 0.0207** | **0.62815 0.0287** |
| **PE 16:0/20:4 (12)** | **0.70981 0.0097** | **-0.66122 0.0192** | 0.50385 0.0949 | **0.64407 0.0238** | **0.68319 0.0143** | 0.41497 0.1798 | **0.79040 0.0022** | **0.63527 0.0264** | **0.63625 0.0261** | 0.46527 0.1275 |
| **PE 16:0/22:6 (12)** | **0.84061 0.0006** | **-0.77280 0.0032** | **0.64473 0.0236** | **0.82943 0.0008** | **0.89969 <.0001** | **0.69791 0.0116** | **0.94216 <.0001** | **0.85221 0.0004** | **0.85543 0.0004** | 0.14460 0.6539 |
| **PE 18:0/18:0 (12)** | **0.58857 0.0441** | -0.46961 0.1235 | **0.58670 0.0449** | **0.52930 0.0768** | **0.58855 0.0441** | **0.65419 0.0210** | 0.56007 0.0582 | **0.58218 0.0470** | 0.52159 0.0820 | -0.55351 0.0619 |
| **PE 18:0/18:1 (12)** | **0.62842 0.0286** | -0.76808 0.0035 | **0.69253 0.0126** | **0.72523 0.0076** | **0.60637 0.0366** | **0.63806 0.0256** | **0.69270 0.0125** | **0.62823 0.0287** | **0.65971 0.0196** | 0.45008 0.1421 |
| **PE 18:0/18:2 (12)** | **0.71725 0.0086** | **-0.81440 0.0013** | **0.77930 0.0028** | **0.73863 0.0061** | **0.58994 0.0435** | **0.59585 0.0409** | **0.66510 0.0183** | **0.64696 0.0230** | **0.60682 0.0364** | **0.67207 0.0167** |
| **PE 18:0/20:4 (12)** | **0.65953 0.0196** | **-0.74793 0.0052** | **0.59806 0.0400** | **0.68841 0.0133** | **0.70706 0.0101** | 0.52049 0.0828 | **0.79939 0.0018** | **0.66449 0.0184** | **0.65068 0.0219** | **0.58692 0.0448** |
| **PE 18:0/22:6 (12)** | **0.85124 0.0004** | **-0.86233 0.0003** | **0.77045 0.0034** | **0.93163 <.0001** | **0.87779 0.0002** | **0.79860 0.0018** | **0.92823 <.0001** | **0.90375 <.0001** | **0.89036 0.0001** | 0.24639 0.4401 |
| **TAG 46:3 FA 12:0 (10)** | 0.55897 0.0930 | **-0.63378 0.0491** | **0.66616 0.0355** | **0.67473 0.0323** | 0.35334 0.3166 | 0.56101 0.0916 | 0.44236 0.2005 | 0.51284 0.1296 | 0.47118 0.1693 | 0.57632 0.0812 |
| **TAG 46:3 FA 16:1 (10)** | **0.68728 0.0281** | **-0.64815 0.0427** | 0.58491 0.0757 | **0.66700 0.0351** | 0.35426 0.3152 | 0.44785 0.1943 | 0.47669 0.1636 | 0.56215 0.0908 | 0.50961 0.1324 | **0.68936 0.0274** |
| **TAG 50:3 FA 18:1 (12)** | 0.40426 0.1924 | **-0.62090 0.0312** | 0.57072 0.0526 | **0.61003 0.0352** | 0.40846 0.1874 | 0.43751 0.1549 | 0.48948 0.1063 | 0.48045 0.1139 | 0.45817 0.1342 | **0.72739 0.0073** |
| **TAG 50:4 FA 14:0 (12)** | 0.34216 0.2763 | -0.56887 0.0536 | **0.59294 0.0422** | **0.59781 0.0401** | 0.42799 0.1651 | 0.45172 0.1404 | 0.48856 0.1070 | 0.45044 0.1417 | 0.43715 0.1553 | **0.58470 0.0458** |
| **TAG 50:4 FA 18:1 (12)** | 0.36200 0.2475 | **-0.58498 0.0457** | 0.55864 0.0590 | **0.58732 0.0447** | 0.36577 0.2423 | 0.42287 0.1708 | 0.44810 0.1440 | 0.44190 0.1503 | 0.42553 0.1679 | **0.68216 0.0145** |
| **TAG 50:4 FA 18:2 (12)** | 0.35808 0.2531 | **-0.58415 0.0461** | **0.59610 0.0408** | **0.60678 0.0364** | 0.44483 0.1473 | 0.46020 0.1322 | 0.50753 0.0921 | 0.46750 0.1254 | 0.45306 0.1391 | **0.60149 0.0385** |
| **TAG 52:1 FA 18:1 (12)** | 0.47323 0.1202 | **-0.61221 0.0343** | 0.51753 0.0848 | **0.57881 0.0486** | 0.30707 0.3316 | 0.40377 0.1930 | 0.42468 0.1688 | 0.47405 0.1195 | 0.45474 0.1375 | **0.72555 0.0076** |
| **TAG 52:2 FA 14:0 (11)** | 0.51595 0.1042 | **-0.66995 0.0241** | 0.60014 0.0509 | **0.61560 0.0438** | 0.42258 0.1954 | 0.45921 0.1554 | 0.55420 0.0769 | 0.51917 0.1017 | 0.51319 0.1064 | **0.70160 0.0161** |
| **TAG 52:2 FA 16:0 (12)** | 0.50500 0.0940 | **-0.67474 0.0161** | 0.57563 0.0502 | **0.64015 0.0249** | 0.39721 0.2011 | 0.47667 0.1172 | 0.49921 0.0985 | 0.53587 0.0725 | 0.51259 0.0884 | **0.72709 0.0074** |
| **TAG 52:2 FA 16:1 (12)** | 0.56061 0.0580 | **-0.62756 0.0289** | 0.47099 0.1222 | **0.58923 0.0438** | 0.34882 0.2665 | 0.36808 0.2391 | 0.47597 0.1178 | 0.53776 0.0713 | 0.49879 0.0988 | **0.77096 0.0033** |
| **TAG 52:2 FA 18:2 (12)** | 0.43382 0.1588 | **-0.60363 0.0377** | 0.56315 0.0566 | **0.57907 0.0485** | 0.33737 0.2835 | 0.41302 0.1821 | 0.43747 0.1550 | 0.45052 0.1416 | 0.43751 0.1549 | **0.70051 0.0112** |
| **TAG 52:3 FA 14:0 (11)** | 0.56507 0.0701 | **-0.69776 0.0170** | **0.60267 0.0497** | **0.64800 0.0311** | 0.53588 0.0893 | 0.46005 0.1545 | 0.65641 0.0283 | 0.56884 0.0678 | 0.55416 0.0769 | **0.69480 0.0176** |
| **TAG 52:3 FA 16:0 (12)** | 0.47506 0.1186 | **-0.67048 0.0170** | **0.62125 0.0311** | **0.66225 0.0190** | 0.45769 0.1346 | 0.50459 0.0943 | 0.54479 0.0670 | 0.53995 0.0700 | 0.52049 0.0828 | **0.66569 0.0181** |
| **TAG 52:3 FA 16:1 (12)** | 0.54367 0.0677 | **-0.70677 0.0102** | 0.53662 0.0721 | **0.64673 0.0230** | 0.46828 0.1247 | 0.47999 0.1143 | 0.55998 0.0583 | **0.60563 0.0369** | 0.55726 0.0598 | **0.79967 0.0018** |
| **TAG 52:3 FA 18:0 (12)** | 0.48969 0.1061 | **-0.61783 0.0323** | 0.54588 0.0664 | **0.59220 0.0425** | 0.34856 0.2668 | 0.39288 0.2065 | 0.46023 0.1322 | 0.48573 0.1094 | 0.46137 0.1311 | **0.74570 0.0054** |
| **TAG 52:3 FA 18:2 (12)** | 0.45728 0.1350 | **-0.66405 0.0185** | **0.61721 0.0325** | **0.65314 0.0213** | 0.44856 0.1436 | 0.50394 0.0948 | 0.53369 0.0739 | 0.52945 0.0767 | 0.51097 0.0896 | **0.66573 0.0181** |
| **TAG 52:3 FA 20:0 (12)** | 0.48654 0.1087 | **-0.67364 0.0163** | 0.61430 0.0336 | **0.65646 0.0204** | 0.46040 0.1320 | 0.48266 0.1120 | 0.55042 0.0637 | 0.54130 0.0691 | 0.51380 0.0875 | **0.69921 0.0114** |
| **TAG 52:3 FA 20:1 (12)** | 0.50249 0.0959 | **-0.65888 0.0198** | 0.56486 0.0557 | **0.66147 0.0191** | 0.50625 0.0931 | 0.46010 0.1323 | 0.61731 0.0325 | 0.56383 0.0562 | 0.56591 0.0551 | **0.67248 0.0166** |
| **TAG 52:3 FA 20:2 (12)** | 0.55781 0.0595 | **-0.63942 0.0252** | 0.48780 0.1077 | **0.61139 0.0347** | 0.44344 0.1488 | 0.33592 0.2857 | 0.56407 0.0561 | 0.53640 0.0722 | 0.51639 0.0856 | **0.79846 0.0018** |
| **TAG 52:4 FA 14:0 (10)** | **0.72401 0.0179** | **-0.83726 0.0025** | **0.67068 0.0338** | **0.75579 0.0114** | 0.55124 0.0986 | **0.68274 0.0296** | **0.66843 0.0346** | **0.72026 0.0188** | **0.71965 0.0190** | **0.71100 0.0212** |
| **TAG 52:4 FA 16:0 (12)** | 0.43749 0.1549 | **-0.62472 0.0299** | **0.62752 0.0289** | **0.64529 0.0234** | 0.45797 0.1343 | 0.47751 0.1164 | 0.53522 0.0729 | 0.50560 0.0936 | 0.48778 0.1077 | **0.60774 0.0361** |
| **TAG 52:4 FA 16:1 (12)** | 0.51044 0.0900 | **-0.69415 0.0123** | **0.59190 0.0426** | **0.67484 0.0161** | 0.54983 0.0640 | 0.51406 0.0873 | **0.62112 0.0311** | **0.62217 0.0307** | 0.57246 0.0517 | **0.71473 0.0090** |
| **TAG 52:4 FA 18:0 (12)** | 0.54297 0.0681 | **-0.59725 0.0403** | **0.57650 0.0497** | **0.63049 0.0280** | 0.36396 0.2448 | 0.38734 0.2135 | 0.47619 0.1176 | 0.52252 0.0814 | 0.47026 0.1229 | **0.66103 0.0193** |
| **TAG 52:4 FA 18:1 (12)** | 0.46380 0.1288 | **-0.67581 0.0158** | **0.60849 0.0358** | **0.66293 0.0188** | 0.48247 0.1121 | 0.50640 0.0930 | 0.56048 0.0580 | 0.56096 0.0578 | 0.52763 0.0779 | **0.69878 0.0115** |
| **TAG 52:4 FA 18:2 (12)** | 0.43968 0.1526 | **-0.63328 0.0271** | **0.63490 0.0265** | **0.64918 0.0224** | 0.48107 0.1133 | 0.49005 0.1058 | 0.55273 0.0623 | 0.51768 0.0847 | 0.49849 0.0990 | **0.61015 0.0351** |
| **TAG 52:4 FA 18:3 (12)** | 0.33971 0.2800 | **-0.57682 0.0496** | 0.57529 0.0503 | **0.58390 0.0462** | 0.34624 0.2702 | 0.43272 0.1600 | 0.41562 0.1791 | 0.41385 0.1811 | 0.39626 0.2022 | **0.62941 0.0283** |
| **TAG 52:4 FA 20:0 (12)** | 0.39600 0.2026 | **-0.60157 0.0385** | **0.62335 0.0303** | **0.62043 0.0314** | 0.43384 0.1588 | 0.47317 0.1203 | 0.49776 0.0996 | 0.47008 0.1230 | 0.45607 0.1362 | **0.58040 0.0479** |
| **TAG 52:5 FA 14:0 (10)** | **0.69380 0.0261** | **-0.81640 0.0040** | **0.68935 0.0274** | **0.76763 0.0095** | 0.51209 0.1302 | **0.68931 0.0274** | 0.61924 0.0562 | **0.70170 0.0237** | **0.68221 0.0298** | **0.66755 0.0349** |
| **TAG 52:5 FA 16:1 (12)** | 0.47725 0.1167 | **-0.63095 0.0278** | **0.61265 0.0342** | **0.67263 0.0165** | 0.55784 0.0595 | 0.48305 0.1117 | **0.61197 0.0344** | **0.59012 0.0434** | 0.53329 0.0742 | **0.59953 0.0394** |
| **TAG 52:5 FA 18:1 (12)** | 0.43481 0.1578 | **-0.60182 0.0384** | **0.61048 0.0350** | **0.65671 0.0203** | 0.45297 0.1392 | 0.47518 0.1185 | 0.51155 0.0891 | 0.54578 0.0664 | 0.48068 0.1137 | **0.59368 0.0418** |
| **TAG 52:5 FA 18:2 (12)** | 0.41496 0.1798 | **-0.61929 0.0318** | **0.62763 0.0289** | **0.64614 0.0232** | 0.49589 0.1011 | 0.49110 0.1049 | 0.55612 0.0604 | 0.52440 0.0801 | 0.49382 0.1027 | **0.58987 0.0435** |
| **TAG 52:5 FA 18:3 (12)** | 0.32067 0.3095 | -0.55510 0.0610 | **0.58943 0.0437** | **0.58262 0.0468** | 0.37140 0.2346 | 0.43478 0.1578 | 0.42963 0.1634 | 0.41311 0.1820 | 0.39300 0.2063 | 0.57562 0.0502 |
| **TAG 52:5 FA 20:3 (11)** | **0.62422 0.0401** | **-0.79156 0.0037** | 0.58698 0.0576 | **0.75153 0.0077** | 0.54620 0.0821 | **0.62701 0.0390** | **0.63621 0.0353** | **0.70133 0.0162** | **0.70483 0.0154** | **0.74495 0.0085** |
| **TAG 52:5 FA 22:5 (12)** | 0.47754 0.1164 | **-0.61361 0.0338** | **0.62664 0.0292** | **0.68174 0.0146** | 0.46819 0.1248 | 0.43570 0.1568 | 0.54175 0.0689 | 0.51601 0.0859 | 0.48369 0.1111 | **0.59822 0.0399** |
| **TAG 53:2 FA 17:0 (12)** | 0.44870 0.1434 | **-0.68336 0.0143** | **0.58821 0.0443** | **0.59126 0.0429** | 0.37816 0.2255 | 0.48865 0.1070 | 0.44406 0.1481 | 0.49019 0.1057 | 0.47101 0.1222 | **0.75290 0.0047** |
| **TAG 53:2 FA 18:1 (12)** | 0.40900 0.1868 | **-0.66151 0.0191** | 0.57165 0.0522 | **0.58533 0.0456** | 0.36683 0.2408 | 0.48560 0.1095 | 0.43764 0.1548 | 0.47119 0.1220 | 0.46109 0.1314 | **0.73558 0.0064** |
| **TAG 53:3 FA 17:0 (12)** | 0.40742 0.1887 | **-0.66846 0.0175** | **0.62749 0.0289** | **0.60697 0.0364** | 0.44156 0.1507 | 0.50161 0.0966 | 0.48623 0.1090 | 0.48166 0.1128 | 0.46217 0.1304 | **0.69131 0.0128** |
| **TAG 53:3 FA 18:2 (12)** | 0.34224 0.2762 | **-0.60571 0.0369** | **0.58860 0.0441** | 0.55647 0.0602 | 0.37826 0.2254 | 0.45632 0.1359 | 0.43133 0.1615 | 0.41915 0.1750 | 0.41522 0.1795 | **0.66668 0.0179** |
| **TAG 53:4 FA 17:0 (12)** | 0.36471 0.2438 | **-0.59893 0.0396** | **0.62445 0.0300** | **0.57674 0.0496** | 0.41593 0.1787 | 0.44783 0.1443 | 0.45692 0.1354 | 0.42847 0.1646 | 0.40797 0.1880 | **0.62573 0.0295** |
| **TAG 53:4 FA 18:2 (12)** | 0.33850 0.2818 | **-0.60671 0.0365** | **0.60867 0.0357** | 0.57321 0.0514 | 0.42908 0.1640 | 0.48004 0.1142 | 0.46900 0.1240 | 0.44686 0.1453 | 0.43244 0.1603 | **0.63124 0.0277** |
| **TAG 54:2 FA 18:0 (12)** | 0.37154 0.2344 | **-0.59530 0.0411** | **0.58092 0.0476** | 0.52769 0.0779 | 0.29766 0.3474 | 0.45244 0.1397 | 0.36630 0.2416 | 0.40045 0.1971 | 0.39658 0.2018 | **0.67014 0.0171** |
| **TAG 54:2 FA 18:1 (12)** | 0.39579 0.2028 | **-0.62847 0.0286** | **0.57870 0.0487** | **0.58179 0.0472** | 0.33957 0.2802 | 0.50865 0.0913 | 0.42946 0.1635 | 0.46609 0.1267 | 0.46375 0.1289 | **0.64226 0.0243** |
| **TAG 54:2 FA 20:1 (12)** | 0.49742 0.0999 | **-0.66702 0.0178** | **0.58028 0.0479** | **0.66046 0.0194** | 0.49936 0.0984 | 0.47905 0.1151 | 0.60559 0.0369 | 0.54928 0.0643 | 0.56221 0.0571 | **0.65743 0.0202** |
| **TAG 54:2 FA 20:2 (12)** | 0.54412 0.0674 | **-0.63104 0.0278** | 0.51350 0.0877 | **0.63149 0.0276** | 0.42795 0.1652 | 0.37690 0.2272 | 0.56019 0.0582 | 0.54002 0.0699 | 0.52620 0.0789 | **0.70323 0.0107** |
| **TAG 54:3 FA 16:0 (12)** | 0.52769 0.0779 | **-0.66184 0.0191** | 0.55434 0.0614 | **0.66214 0.0190** | 0.51906 0.0838 | 0.43501 0.1576 | **0.62998 0.0281** | 0.56619 0.0550 | 0.56670 0.0547 | **0.67711 0.0156** |
| **TAG 54:3 FA 16:1 (12)** | **0.61434 0.0443** | **-0.73996 0.0092** | **0.60640 0.0479** | **0.66333 0.0261** | 0.52707 0.0957 | 0.50091 0.1165 | **0.66253 0.0263** | **0.61501 0.0440** | 0.59476 0.0536 | **0.74314 0.0088** |
| **TAG 54:3 FA 20:1 (12)** | 0.46858 0.1244 | **-0.64357 0.0239** | 0.57542 0.0503 | **0.64264 0.0242** | 0.54046 0.0697 | 0.47650 0.1173 | **0.63427 0.0267** | 0.53927 0.0704 | 0.56334 0.0565 | **0.60262 0.0381** |
| **TAG 54:3 FA 20:2 (12)** | 0.55833 0.0592 | **-0.68542 0.0139** | 0.55079 0.0635 | **0.67759 0.0155** | 0.55052 0.0636 | 0.44465 0.1475 | **0.66225 0.0190** | **0.59793 0.0400** | **0.59436 0.0415** | **0.69170 0.0127** |
| **TAG 54:4 FA 16:1 (12)** | 0.47838 0.1157 | **-0.65655 0.0204** | 0.54018 0.0698 | **0.63346 0.0270** | 0.53549 0.0728 | 0.44587 0.1463 | 0.63192 0.0275 | 0.56452 0.0558 | 0.55647 0.0602 | **0.72361 0.0078** |
| **TAG 54:4 FA 20:1 (12)** | 0.47913 0.1150 | **-0.63797 0.0256** | **0.58215 0.0470** | **0.65158 0.0217** | 0.53229 0.0748 | 0.46892 0.1241 | **0.63124 0.0277** | 0.54897 0.0645 | 0.55705 0.0599 | **0.61760 0.0324** |
| **TAG 54:4 FA 20:2 (12)** | 0.55271 0.0624 | **-0.67531 0.0160** | 0.56586 0.0551 | **0.67989 0.0150** | **0.59367 0.0418** | 0.45304 0.1391 | **0.69292 0.0125** | **0.60311 0.0379** | **0.60451 0.0373** | **0.63750 0.0257** |
| **TAG 54:5 FA 20:2 (12)** | 0.48886 0.1068 | **-0.64065 0.0248** | 0.54383 0.0676 | **0.64884 0.0224** | 0.53225 0.0749 | 0.43451 0.1581 | 0.63156 0.0276 | 0.56783 0.0541 | 0.55959 0.0585 | **0.65486 0.0208** |
| **TAG 54:5 FA 22:4 (12)** | **0.66032 0.0377** | **-0.76197 0.0104** | 0.56150 0.0912 | **0.66531 0.0358** | 0.40952 0.2399 | 0.55864 0.0932 | 0.54247 0.1052 | 0.62962 0.0511 | 0.61953 0.0561 | **0.78658 0.0070** |
| **TAG 54:5 FA 22:5 (12)** | **0.59251 0.0423** | **-0.61346 0.0339** | 0.51852 0.0841 | **0.65535 0.0207** | 0.38941 0.2109 | 0.37289 0.2326 | 0.51392 0.0874 | 0.55381 0.0617 | 0.51303 0.0881 | **0.64532 0.0234** |
| **TAG 54:6 FA 22:5 (12)** | **0.62680 0.0292** | **-0.68461 0.0140** | **0.62239 0.0307** | **0.77517 0.0031** | 0.57192 0.0520 | 0.48183 0.1127 | **0.64595 0.0233** | **0.65804 0.0200** | **0.59874 0.0397** | **0.59620 0.0408** |
| **TAG 54:6 FA 22:6 (12)** | **0.61362 0.0338** | -0.55652 0.0602 | **0.68057 0.0149** | **0.77224 0.0032** | 0.52134 0.0822 | 0.57453 0.0507 | 0.55357 0.0619 | **0.65117 0.0218** | 0.54821 0.0650 | 0.08014 0.8045 |
| **TAG 54:7 FA 22:5 (12)** | 0.46271 0.1298 | -0.57320 0.0514 | **0.66740 0.0177** | **0.72651 0.0074** | **0.61157 0.0346** | 0.54842 0.0648 | **0.63637 0.0261** | **0.60105 0.0387** | 0.55142 0.0631 | 0.30453 0.3358 |
| **TAG 54:7 FA 22:6 (12)** | 0.51958 0.0834 | -0.44279 0.1494 | **0.62457 0.0299** | **0.70114 0.0111** | 0.54119 0.0692 | 0.53749 0.0715 | 0.51468 0.0869 | 0.60552 0.0369 | 0.48195 0.1126 | -0.10712 0.7404 |
| **TAG 56:3 FA 18:0 (12)** | 0.48034 0.1140 | **-0.62272 0.0306** | **0.58765 0.0445** | **0.60002 0.0392** | 0.39487 0.2040 | 0.42490 0.1685 | 0.49876 0.0988 | 0.48167 0.1128 | 0.47694 0.1169 | **0.68239 0.0145** |
| **TAG 56:3 FA 20:2 (12)** | 0.51229 0.0886 | **-0.68113 0.0147** | 0.57472 0.0506 | **0.64485 0.0236** | 0.47153 0.1217 | 0.46002 0.1324 | **0.58107 0.0475** | 0.55204 0.0627 | 0.54972 0.0641 | **0.72373 0.0078** |
| **TAG 56:4 FA 16:0 (12)** | 0.48061 0.1137 | **-0.62828 0.0287** | 0.51768 0.0847 | **0.61641 0.0328** | 0.45115 0.1410 | 0.40848 0.1874 | 0.57524 0.0504 | 0.51194 0.0888 | 0.53207 0.0750 | **0.66704 0.0178** |
| **TAG 56:4 FA 18:0 (12)** | 0.44640 0.1458 | **-0.61636 0.0328** | **0.59284 0.0422** | **0.61286 0.0341** | 0.40490 0.1917 | 0.41585 0.1788 | 0.48444 0.1105 | 0.46703 0.1258 | 0.44621 0.1459 | **0.66148 0.0191** |
| **TAG 56:4 FA 18:1 (12)** | 0.37202 0.2337 | **-0.60598 0.0368** | 0.53226 0.0748 | **0.58561 0.0454** | 0.46106 0.1314 | 0.45953 0.1329 | 0.55731 0.0598 | 0.47937 0.1148 | 0.49655 0.1006 | **0.62970 0.0282** |
| **TAG 56:4 FA 20:2 (12)** | 0.38400 0.2178 | **-0.61709 0.0326** | 0.51056 0.0899 | **0.59130 0.0429** | 0.52329 0.0808 | 0.47140 0.1219 | 0.61590 0.0330 | 0.51337 0.0878 | 0.54617 0.0662 | **0.60382 0.0376** |
| **TAG 56:5 FA 18:0 (12)** | 0.43786 0.1546 | **-0.67340 0.0164** | 0.72825 0.0072 | **0.70749 0.0101** | **0.57684 0.0496** | **0.60995 0.0352** | **0.58721 0.0447** | **0.55116 0.0633** | **0.52127 0.0822** | 0.40507 0.1915 |
| **TAG 56:5 FA 20:2 (12)** | 0.43244 0.1603 | **-0.61187 0.0345** | 0.53941 0.0703 | **0.62076 0.0312** | **0.59316 0.0421** | 0.46902 0.1240 | **0.67414 0.0162** | 0.54755 0.0654 | 0.56731 0.0544 | 0.53579 0.0726 |
| **TAG 56:5 FA 20:4 (12)** | 0.34945 0.2655 | **-0.62415 0.0301** | **0.68867 0.0133** | **0.64226 0.0243** | 0.56976 0.0531 | **0.59197 0.0426** | 0.55861 0.0590 | 0.49156 0.1046 | 0.47564 0.1181 | 0.32455 0.3034 |
| **TAG 56:5 FA 22:5 (12)** | 0.54745 0.0654 | **-0.73577 0.0064** | **0.67917 0.0151** | **0.76072 0.0041** | 0.54500 0.0669 | **0.63709 0.0259** | **0.61369 0.0338** | **0.64064 0.0248** | **0.62453 0.0299** | 0.47609 0.1177 |
| **TAG 56:6 FA 16:0 (12)** | 0.43657 0.1559 | **-0.69807 0.0116** | **0.67705 0.0156** | **0.71116 0.0095** | 0.51373 0.0876 | **0.65242 0.0215** | 0.52802 0.0776 | 0.58456 0.0459 | 0.55628 0.0603 | 0.41623 0.1783 |
| **TAG 56:6 FA 18:0 (12)** | 0.42175 0.1721 | -0.57342 0.0513 | **0.75234 0.0048** | **0.69866 0.0115** | 0.57720 0.0494 | **0.58522 0.0456** | 0.55824 0.0592 | 0.53631 0.0723 | 0.46489 0.1278 | 0.19991 0.5333 |
| **TAG 56:6 FA 18:1 (12)** | 0.35481 0.2578 | **-0.67256 0.0166** | **0.61527 0.0332** | **0.65902 0.0198** | 0.50978 0.0904 | **0.61750 0.0324** | 0.53981 0.0701 | 0.54314 0.0680 | 0.54048 0.0696 | 0.44251 0.1497 |
| **TAG 56:6 FA 18:3 (11)** | 0.50846 0.1103 | **-0.78200 0.0045** | **0.70154 0.0161** | **0.77061 0.0055** | **0.68170 0.0209** | **0.79487 0.0035** | **0.69941 0.0166** | **0.67951 0.0215** | **0.70489 0.0154** | 0.44268 0.1727 |
| **TAG 56:6 FA 20:2 (10)** | 0.46185 0.1790 | **-0.69252 0.0264** | **0.63841 0.0470** | **0.74444 0.0135** | **0.71764 0.0195** | **0.75990 0.0108** | **0.71522 0.0201** | **0.64732 0.0430** | **0.70931 0.0216** | 0.38141 0.2768 |
| **TAG 56:6 FA 22:4 (12)** | 0.39292 0.2064 | **-0.60099 0.0388** | 0.50631 0.0930 | **0.58709 0.0448** | 0.42704 0.1662 | 0.40279 0.1942 | 0.52885 0.0771 | 0.47318 0.1203 | 0.48632 0.1089 | **0.67451 0.0161** |
| **TAG 56:6 FA 22:5 (12)** | 0.59540 0.0411 | **-0.80082 0.0017** | **0.77217 0.0032** | **0.86267 0.0003** | **0.71800 0.0085** | **0.75861 0.0042** | **0.71772 0.0086** | **0.74191 0.0057** | **0.69653 0.0118** | 0.32924 0.2960 |
| **TAG 56:6 FA 22:6 (12)** | 0.58879 0.0440 | **-0.57796 0.0490** | **0.73866 0.0061** | **0.78448 0.0025** | **0.58511 0.0457** | **0.69654 0.0118** | 0.56542 0.0554 | **0.67843 0.0153** | 0.57548 0.0502 | -0.10479 0.7459 |
| **TAG 56:7 FA 16:0 (12)** | 0.46883 0.1242 | **-0.58190 0.0472** | **0.74844 0.0051** | **0.75601 0.0044** | **0.58745 0.0446** | **0.67815 0.0154** | 0.55404 0.0616 | **0.61957 0.0317** | 0.52349 0.0807 | 0.01106 0.9728 |
| **TAG 56:7 FA 16:1 (11)** | 0.35692 0.2813 | **-0.66375 0.0259** | **0.66157 0.0266** | **0.64105 0.0335** | **0.63873 0.0344** | **0.73807 0.0095** | **0.61918 0.0422** | **0.60729 0.0475** | 0.56308 0.0713 | 0.15304 0.6533 |
| **TAG 56:7 FA 18:1 (12)** | 0.37100 0.2351 | -0.54614 0.0662 | **0.69628 0.0119** | **0.70213 0.0109** | **0.58433 0.0460** | **0.66419 0.0185** | 0.55536 0.0609 | 0.57051 0.0527 | 0.51313 0.0880 | -0.01586 0.9610 |
| **TAG 56:7 FA 20:3 (11)** | 0.35296 0.2870 | **-0.67050 0.0239** | 0.54108 0.0856 | **0.64401 0.0325** | **0.60374 0.0492** | **0.70731 0.0149** | **0.61747 0.0430** | 0.56919 0.0676 | **0.64795 0.0311** | 0.30760 0.3575 |
| **TAG 56:7 FA 22:5 (12)** | 0.54073 0.0695 | **-0.70344 0.0107** | **0.74104 0.0058** | **0.80906 0.0014** | **0.68847 0.0133** | **0.65705 0.0203** | **0.70371 0.0107** | **0.68286 0.0144** | **0.62789 0.0288** | 0.31993 0.3107 |
| **TAG 56:7 FA 22:6 (12)** | 0.50378 0.0949 | -0.46197 0.1305 | **0.67920 0.0151** | **0.70884 0.0099** | **0.59413 0.0416** | **0.64675 0.0230** | 0.53049 0.0760 | **0.61538 0.0332** | 0.49415 0.1025 | -0.31394 0.3203 |
| **TAG 56:8 FA 22:5 (12)** | 0.46962 0.1235 | **-0.58228 0.0470** | **0.70833 0.0099** | **0.75332 0.0047** | **0.66381 0.0186** | **0.60330 0.0378** | **0.64424 0.0237** | **0.62338 0.0303** | 0.54433 0.0673 | 0.13600 0.6734 |
| **TAG 58:5 FA 18:1 (11)** | **0.61191 0.0454** | **-0.81338 0.0023** | **0.65457 0.0289** | **0.77882 0.0047** | **0.67927 0.0215** | **0.73122 0.0106** | **0.74773 0.0081** | **0.72384 0.0118** | **0.76495 0.0061** | **0.60615 0.0481** |
| **TAG 58:6 FA 18:1 (12)** | 0.44015 0.1522 | **-0.66743 0.0177** | **0.60493 0.0372** | **0.67124 0.0169** | **0.65004 0.0221** | 0.57386 0.0510 | **0.69210 0.0126** | **0.58510 0.0457** | **0.61737 0.0324** | 0.44608 0.1461 |
| **TAG 58:6 FA 22:5 (12)** | 0.51566 0.0862 | **-0.72899 0.0071** | **0.77760 0.0029** | **0.81806 0.0011** | **0.69729 0.0117** | **0.77556 0.0030** | **0.69067 0.0129** | **0.69763 0.0117** | **0.66530 0.0182** | 0.19179 0.5504 |
| **TAG 58:7 FA 18:2 (12)** | 0.33931 0.2806 | -0.53317 0.0743 | **0.59904 0.0396** | **0.60823 0.0359** | **0.62307 0.0304** | 0.54216 0.0686 | **0.62133 0.0310** | 0.52621 0.0788 | 0.52380 0.0805 | 0.21337 0.5055 |
| **TAG 58:7 FA 20:4 (10)** | 0.46254 0.1783 | **-0.68953 0.0274** | 0.58283 0.0770 | **0.66395 0.0363** | **0.73116 0.0163** | **0.75824 0.0110** | **0.72737 0.0171** | 0.63082 0.0505 | **0.71107 0.0211** | 0.08540 0.8145 |
| **TAG 58:7 FA 22:5 (12)** | 0.50474 0.0942 | **-0.65029 0.0220** | **0.74193 0.0057** | **0.78978 0.0022** | **0.76664 0.0036** | **0.75518 0.0045** | **0.74815 0.0051** | **0.68330 0.0143** | **0.66437 0.0184** | 0.00283 0.9930 |
| **TAG 58:7 FA 22:6 (12)** | 0.42196 0.1718 | -0.42428 0.1692 | **0.68200 0.0146** | **0.62730 0.0290** | 0.52447 0.0800 | 0.67230 0.0166 | 0.45301 0.1392 | 0.54823 0.0650 | 0.45195 0.1402 | -0.35268 0.2608 |
| **TAG 58:8 FA 22:5 (12)** | 0.44021 0.1521 | -0.56136 0.0575 | **0.63022 0.0280** | **0.70758 0.0100** | **0.73883 0.0061** | **0.62642 0.0293** | **0.73948 0.0060** | **0.62188 0.0309** | **0.60397 0.0375** | 0.02377 0.9415 |

**Supplemental Table 2.** Metabolites with nominally significant r-values in CSF, ranging from -1 to 1. p-values are listed beneath r-values. p-values < 0.05 are in Bold. (N) represents the number of participants for whom circulating metabolite levels were determined.

|  | **TFC** | **TMS** | **FA** | **INDEP** | **SCOLOR** | **SWORD** | **SINTERF** | **VERBAL** | **SDMT** | **BEHAV** |
| --- | --- | --- | --- | --- | --- | --- | --- | --- | --- | --- |
| **NAD^+^ (12)** | **-0.63301 0.0271** | 0.55627 0.0603 | **-0.63015 0.0281** | **-0.62093 0.0312** | **-0.58662 0.0450** | -0.52124 0.0822 | -0.56691 0.0546 | **-0.58915 0.0438** | -0.45860 0.1337 | -0.33312 0.2900 |
| **Arg (12)** | -0.57077 0.0526 | 0.52893 0.0770 | -0.42381 0.1698 | -0.42052 0.1735 | -0.38252 0.2197 | -0.49895 0.0987 | -0.45159 0.1406 | -0.45780 0.1345 | -0.46849 0.1245 | -0.10442 0.7467 |
| **Asn (11)** | 0.71603 0.0132 | -0.58218 0.0602 | 0.56565 0.0697 | **0.70183 0.0161** | 0.48505 0.1305 | 0.39172 0.2335 | 0.46918 0.1454 | **0.61347 0.0447** | 0.43019 0.1866 | 0.31258 0.3493 |
| **CREAT (12)** | **-0.63298 0.0272** | **0.68774 0.0134** | **-0.61744 0.0324** | **-0.59082 0.0431** | **-0.67766 0.0155** | **-0.59284 0.0422** | **-0.57991 0.0481** | -0.54004 0.0699 | -0.48952 0.1062 | 0.02572 0.9368 |
| **DAG (12)** | -0.33242 0.2911 | 0.52714 0.0782 | -0.49849 0.0990 | **-0.59357 0.0419** | **-0.66127 0.0192** | -0.56941 0.0533 | **-0.65056 0.0220** | -0.56748 0.0543 | -0.54096 0.0693 | -0.29163 0.3577 |
| **FFA (12)** | -0.52214 0.0816 | 0.38835 0.2122 | -0.54435 0.0673 | **-0.63314 0.0271** | -0.52388 0.0804 | -0.43204 0.160712 | -0.54454 0.0672 | -0.51234 0.0886 | -0.42926 0.1638 | 0.16105 0.6170 |
| **DAG 16:0/16:0 (12)** | -0.45307 0.1391 | **0.58354 0.0464** | -0.55764 0.0596 | **-0.61035 0.0351** | **-0.62178 0.0309** | **-0.59358 0.0419** | **-0.70686 0.0102** | **-0.62521 0.0297** | -0.57209 0.0519 | -0.34515 0.2719 |
| **FFA 12:0 (12)** | -0.55722 0.0598 | 0.56533 0.0554 | **-0.58708 0.0448** | **-0.61441 0.0335** | -0.49253 0.1038 | -0.51956 0.0834 | -0.48847 0.1071 | -0.57105 0.0525 | -0.36744 0.2400 | -0.16311 0.6125 |
| **FFA 14:0 (12)** | -0.39069 0.2092 | 0.37934 0.2239 | -0.30525 0.3346 | -0.53377 0.0739 | -0.47349 0.1200 | -0.43615 0.1564 | -0.55601 0.0605 | -0.56313 0.0566 | -0.51697 0.0852 | 0.05440 0.8667 |
| **FFA 16:0 (12)** | -0.51748 0.0849 | 0.39320 0.2061 | -0.49324 0.1032 | **-0.62765 0.0289** | -0.53744 0.0715 | -0.43493 0.1577 | -0.54027 0.0698 | -0.53475 0.0732 | -0.45731 0.1350 | 0.20890 0.5147 |
| **FFA 17:0 (12)** | -0.49004 0.1058 | 0.44861 0.1435 | -0.48439 0.1105 | **-0.61374 0.0338** | -0.49734 0.0999 | -0.50145 0.0967 | -0.54973 0.0641 | -0.56885 0.0536 | -0.49165 0.1045 | 0.07652 0.8132 |
| **FFA 18:0 (12)** | **-0.60541 0.0370** | 0.44879 0.1433 | **-0.68565 0.0138** | **-0.62598 0.0294** | -0.48397 0.1109 | -0.43106 0.1618 | -0.50692 0.0926 | -0.48908 0.1066 | -0.40743 0.1886 | 0.04533 0.8887 |
| **FFA 20:5 (12)** | -0.50959 0.0906 | 0.56062 0.0579 | **-0.60801 0.0360** | -0.54643 0.0660 | -0.42540 0.1680 | -0.53047 0.0760 | -0.42096 0.1730 | -0.48390 0.1109 | -0.39310 0.2062 | -0.12829 0.6911 |
| **FFA 22:4 (12)** | -0.55721 0.0598 | 0.46295 0.1296 | **-0.60172 0.0385** | **-0.62137 0.0310** | -0.41631 0.1783 | -0.29929 0.3446 | -0.44880 0.1433 | -0.41014 0.1854 | -0.32768 0.2985 | -0.16973 0.5979 |
| **FFA22:5 (12)** | **-0.62081 0.0312** | 0.49922 0.0985 | **-0.65311 0.0213** | **-0.67744 0.0155** | **-0.58142 0.0474** | -0.34463 0.2726 | **-0.59175 0.0427** | -0.46437 0.1283 | -0.38207 0.2203 | -0.16169 0.6156 |
| **TAG 36:0 FA 12:0 (12)** | -0.45617 0.1361 | **0.64705 0.0229** | -0.44372 0.1485 | **-0.58828 0.0442** | **-0.61114 0.0348** | **-0.72256 0.0079** | **-0.65462 0.0209** | **-0.71781 0.0086** | **-0.76483 0.0038** | -0.09571 0.7673 |
| **TAG 38:0 FA 12:0 (12)** | -0.39081 0.2091 | 0.55640 0.0603 | -0.34927 0.2658 | -0.46696 0.1259 | -0.55184 0.0629 | **-0.66171 0.0191** | **-0.60324 0.0378** | **-0.64588 0.0233** | **-0.69252 0.0126** | -0.03066 0.9246 |
| **TAG 40:0 FA 14:0 (10)** | -0.40157 0.2500 | 0.57069 0.0849 | -0.22955 0.5235 | -0.39792 0.2548 | -0.54923 0.1001 | -0.60043 0.0664 | **-0.64789 0.0428** | **-0.65251 0.0408** | **-0.84968 0.0019** | 0.07679 0.8330 |
| **TAG 42:0 FA 14:0 (10)** | -0.27284 0.4456 | 0.47678 0.1635 | -0.15079 0.6775 | -0.47462 0.1657 | -0.61589 0.0580 | -0.58585 0.0751 | **-0.69041 0.0271** | **-0.66371 0.0364** | **-0.76127 0.0105** | 0.14264 0.6942 |
| **TAG 48:0 FA 16:0 (12)** | -0.18371 0.5676 | 0.32793 0.2981 | -0.17402 0.5886 | -0.45825 0.1341 | -0.55084 0.0634 | -0.52811 0.0776 | -0.55787 0.0595 | -0.52523 0.0795 | -0.55670 0.0601 | 0.24393 0.4448 |
| **TAG 48:0 FA 18:0 (11)** | -0.22411 0.5077 | 0.41542 0.2039 | -0.23836 0.4803 | -0.51677 0.1036 | -0.53866 0.0873 | **-0.61985 0.0419** | -0.59658 0.0527 | -0.60012 0.0509 | -**0.62052 0.0416** | 0.04427 0.8972 |
| **TAG 49:0 FA 17:0 (12)** | -0.22231 0.4874 | 0.33565 0.2861 | -0.14720 0.6480 | -0.45835 0.1340 | -0.56278 0.0568 | -0.48793 0.1076 | -**0.58055 0.0478** | -0.54712 0.0656 | -**0.61203 0.0344** | 0.19965 0.5339 |
| **TAG 52:0 FA 18:0 (12)** | -0.49528 0.1016 | 0.52590 0.0791 | **-0.81643 0.0012** | **-0.63264 0.0273** | -0.54299 0.0681 | **-0.61278 0.0341** | -0.48569 0.1094 | -0.44103 0.1512 | -0.30903 0.3284 | -0.01946 0.9521 |
| **PC 16:0/16:0 (12)** | **-0.63353 0.0270** | **0.69482 0.0121** | **-0.75665 0.0044** | **-0.79430 0.0020** | **-0.73172 0.0068** | **-0.78416 0.0025** | **-0.71805 0.0085** | **-0.70028 0.0112** | **-0.69703 0.0118** | -0.05013 0.8770 |
| **PC 16:0/18:1 (12)** | -0.50344 0.0952 | **0.66836 0.0175** | **-0.80431 0.0016** | **-0.71116 0.0095** | **-0.65226 0.0215** | **-0.75579 0.0045** | -0.52704 0.0783 | -0.55175 0.0629 | -0.50044 0.0975 | -0.00208 0.9949 |
| **SM 14:0 (12)** | -0.46373 0.1289 | **0.59964 0.0393** | **-0.72958 0.0071** | **-0.63653 0.0260** | **-0.66047 0.0194** | **-0.73856 0.0061** | -0.54830 0.0649 | -0.53659 0.0721 | -0.51292 0.0881 | 0.04225 0.8963 |
| **SM 16:0 (12)** | -0.54670 0.0659 | 0.56956 0.0532 | **-0.80079 0.0017** | **-0.60766 0.0361** | -0.52014 0.0830 | **-0.61982 0.0316** | -0.43000 0.1629 | -0.42318 0.1705 | -0.32295 0.3059 | 0.01414 0.9652 |
| **SM 18:0 (12)** | **-0.58265 0.0468** | **0.70786 0.0100** | **-0.81861 0.0011** | **-0.71406 0.0091** | **-0.67289 0.0165** | **-0.78165 0.0027** | **-0.61777 0.0323** | **-0.61816 0.0322** | **-0.59467 0.0414** | -0.14129 0.6614 |
| **SM 18:1 (12)** | -0.54474 0.0670 | **0.60080 0.0388** | -0.78496 0.0025 | **-0.67204 0.0167** | -0.55979 0.0584 | **-0.66257 0.0189** | -0.49749 0.0998 | -0.49145 0.1047 | -0.44066 0.1516 | -0.06595 0.8386 |
| **SM 20:1 (11)** | **-0.61910 0.0422** | 0.44141 0.1741 | -0.77440 0.0051 | -0.59126 0.0554 | -0.46344 0.1511 | -0.50316 0.1146 | -0.38855 0.2376 | -0.41079 0.2095 | -0.24749 0.4631 | 0.07163 0.8342 |
| **PC (12)** | -0.47975 0.1145 | 0.57458 0.0507 | **-0.78322 0.0026** | **-0.68639 0.0137** | -0.56516 0.0555 | **-0.68189 0.0146** | -0.45697 0.1353 | -0.48197 0.1126 | -0.39102 0.2088 | 0.02970 0.9270 |

**Supplemental Figure 1. Associations with Clinical Outcomes for TAG Species in plasma**


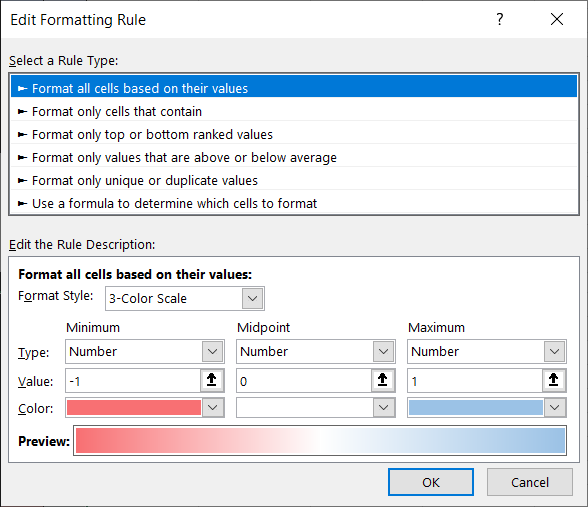


**-1**

**1**

**(r-values ranging from -1 (red) to 1 (blue))**

|  | **TFC** | **TMS** | **FA** | **IND** | **SCo** | **SWo** | **SIn** | **Ver** | **SDMT** | **Beh** |
| --- | --- | --- | --- | --- | --- | --- | --- | --- | --- | --- |
| **TAG 46:3 FA 12:0 (10)** | _1_ | _-1_ | _1_ | _1_ | _0_ | _1_ | _0_ | _1_ | _0_ | _1_ |
| **TAG 46:3 FA 16:1 (10)** | _1_ | _-1_ | _1_ | _1_ | _0_ | _0_ | _0_ | _1_ | _1_ | _1_ |
| **TAG 48:4 FA 14:1 (10)** | _1_ | _-1_ | _1_ | _1_ | _0_ | _0_ | _0_ | _1_ | _0_ | _1_ |
| **TAG 50:3 FA 18:1 (12)** | _0_ | _-1_ | _1_ | _1_ | _0_ | _0_ | _0_ | _0_ | _0_ | _1_ |
| **TAG 50:4 FA 14:0 (12)** | _0_ | _-1_ | _1_ | _1_ | _0_ | _0_ | _0_ | _0_ | _0_ | _1_ |
| **TAG 50:4 FA 18:1 (12)** | _0_ | _-1_ | _1_ | _1_ | _0_ | _0_ | _0_ | _0_ | _0_ | _1_ |
| **TAG 50:4 FA 18:2 (12)** | _0_ | _-1_ | _1_ | _1_ | _0_ | _0_ | _1_ | _0_ | _0_ | _1_ |
| **TAG 52:1 FA 18:1 (12)** | _0_ | _-1_ | _1_ | _1_ | _0_ | _0_ | _0_ | _0_ | _0_ | _1_ |
| **TAG 52:2 FA 14:0 (11)** | _1_ | _-1_ | _1_ | _1_ | _0_ | _0_ | _1_ | _1_ | _1_ | _1_ |
| **TAG 52:2 FA 16:0 (12)** | _1_ | _-1_ | _1_ | _1_ | _0_ | _0_ | _0_ | _1_ | _1_ | _1_ |
| **TAG 52:2 FA 16:1 (12)** | _1_ | _-1_ | _0_ | _1_ | _0_ | _0_ | _0_ | _1_ | _0_ | _1_ |
| **TAG 52:2 FA 18:2 (12)** | _0_ | _-1_ | _1_ | _1_ | _0_ | _0_ | _0_ | _0_ | _0_ | _1_ |
| **TAG 52:3 FA 14:0 (11)** | _1_ | _-1_ | _1_ | _1_ | _1_ | _0_ | _1_ | _1_ | _1_ | _1_ |
| **TAG 52:3 FA 16:0 (12)** | _0_ | _-1_ | _1_ | _1_ | _0_ | _1_ | _1_ | _1_ | _1_ | _1_ |
| **TAG 52:3 FA 16:1 (12)** | _1_ | _-1_ | _1_ | _1_ | _0_ | _0_ | _1_ | _1_ | _1_ | _1_ |
| **TAG 52:3 FA 18:0 (12)** | _0_ | _-1_ | _1_ | _1_ | _0_ | _0_ | _0_ | _0_ | _0_ | _1_ |
| **TAG 52:3 FA 18:2 (12)** | _0_ | _-1_ | _1_ | _1_ | _0_ | _1_ | _1_ | _1_ | _1_ | _1_ |
| **TAG 52:3 FA 20:0 (12)** | _0_ | _-1_ | _1_ | _1_ | _0_ | _0_ | _1_ | _1_ | _1_ | _1_ |
| **TAG 52:3 FA 20:1 (12)** | _1_ | _-1_ | _1_ | _1_ | _1_ | _0_ | _1_ | _1_ | _1_ | _1_ |
| **TAG 52:3 FA 20:2 (12)** | _1_ | _-1_ | _0_ | _1_ | _0_ | _0_ | _1_ | _1_ | _1_ | _1_ |
| **TAG 52:4 FA 14:0 (10)** | _1_ | _-1_ | _1_ | _1_ | _1_ | _1_ | _1_ | _1_ | _1_ | _1_ |
| **TAG 52:4 FA 16:0 (12)** | _0_ | _-1_ | _1_ | _1_ | _0_ | _0_ | _1_ | _1_ | _0_ | _1_ |
| **TAG 52:4 FA 16:1 (12)** | _1_ | _-1_ | _1_ | _1_ | _1_ | _1_ | _1_ | _1_ | _1_ | _1_ |
| **TAG 52:4 FA 18:0 (12)** | _1_ | _-1_ | _1_ | _1_ | _0_ | _0_ | _0_ | _1_ | _0_ | _1_ |
| **TAG 52:4 FA 18:1 (12)** | _0_ | _-1_ | _1_ | _1_ | _0_ | _1_ | _1_ | _1_ | _1_ | _1_ |
| **TAG 52:4 FA 18:2 (12)** | _0_ | _-1_ | _1_ | _1_ | _0_ | _0_ | _1_ | _1_ | _0_ | _1_ |
| **TAG 52:4 FA 18:3 (12)** | _0_ | _-1_ | _1_ | _1_ | _0_ | _0_ | _0_ | _0_ | _0_ | _1_ |
| **TAG 52:4 FA 20:0 (12)** | _0_ | _-1_ | _1_ | _1_ | _0_ | _0_ | _0_ | _0_ | _0_ | _1_ |
| **TAG 52:5 FA 14:0 (10)** | _1_ | _-1_ | _1_ | _1_ | _1_ | _1_ | _1_ | _1_ | _1_ | _1_ |
| **TAG 52:5 FA 16:1 (12)** | _0_ | _-1_ | _1_ | _1_ | _1_ | _0_ | _1_ | _1_ | _1_ | _1_ |
| **TAG 52:5 FA 18:1 (12)** | _0_ | _-1_ | _1_ | _1_ | _0_ | _0_ | _1_ | _1_ | _0_ | _1_ |
| **TAG 52:5 FA 18:2 (12)** | _0_ | _-1_ | _1_ | _1_ | _0_ | _0_ | _1_ | _1_ | _0_ | _1_ |
| **TAG 52:5 FA 18:3 (12)** | _0_ | _-1_ | _1_ | _1_ | _0_ | _0_ | _0_ | _0_ | _0_ | _1_ |
| **TAG 52:5 FA 20:3 (11)** | _1_ | _-1_ | _1_ | _1_ | _1_ | _1_ | _1_ | _1_ | _1_ | _1_ |
| **TAG 52:5 FA 22:5 (12)** | _0_ | _-1_ | _1_ | _1_ | _0_ | _0_ | _1_ | _1_ | _0_ | _1_ |
| **TAG 53:2 FA 17:0 (12)** | _0_ | _-1_ | _1_ | _1_ | _0_ | _0_ | _0_ | _0_ | _0_ | _1_ |
| **TAG 53:2 FA 18:1 (12)** | _0_ | _-1_ | _1_ | _1_ | _0_ | _0_ | _0_ | _0_ | _0_ | _1_ |
| **TAG 53:3 FA 17:0 (12)** | _0_ | _-1_ | _1_ | _1_ | _0_ | _1_ | _0_ | _0_ | _0_ | _1_ |
| **TAG 53:3 FA 18:2 (12)** | _0_ | _-1_ | _1_ | _1_ | _0_ | _0_ | _0_ | _0_ | _0_ | _1_ |
| **TAG 53:4 FA 17:0 (12)** | _0_ | _-1_ | _1_ | _1_ | _0_ | _0_ | _0_ | _0_ | _0_ | _1_ |
| **TAG 53:4 FA 18:2 (12)** | _0_ | _-1_ | _1_ | _1_ | _0_ | _0_ | _0_ | _0_ | _0_ | _1_ |
| **TAG 54:2 FA 18:0 (12)** | _0_ | _-1_ | _1_ | _1_ | _0_ | _0_ | _0_ | _0_ | _0_ | _1_ |
| **TAG 54:2 FA 18:1 (12)** | _0_ | _-1_ | _1_ | _1_ | _0_ | _1_ | _0_ | _0_ | _0_ | _1_ |
| **TAG 54:2 FA 20:1 (12)** | _0_ | _-1_ | _1_ | _1_ | _0_ | _0_ | _1_ | _1_ | _1_ | _1_ |
| **TAG 54:2 FA 20:2 (12)** | _1_ | _-1_ | _1_ | _1_ | _0_ | _0_ | _1_ | _1_ | _1_ | _1_ |
| **TAG 54:3 FA 16:0 (12)** | _1_ | _-1_ | _1_ | _1_ | _1_ | _0_ | _1_ | _1_ | _1_ | _1_ |
| **TAG 54:3 FA 16:1 (12)** | _1_ | _-1_ | _1_ | _1_ | _1_ | _1_ | _1_ | _1_ | _1_ | _1_ |
| **TAG 54:3 FA 20:1 (12)** | _0_ | _-1_ | _1_ | _1_ | _1_ | _0_ | _1_ | _1_ | _1_ | _1_ |
| **TAG 54:3 FA 20:2 (12)** | _1_ | _-1_ | _1_ | _1_ | _1_ | _0_ | _1_ | _1_ | _1_ | _1_ |
| **TAG 54:4 FA 16:1 (12)** | _0_ | _-1_ | _1_ | _1_ | _1_ | _0_ | _1_ | _1_ | _1_ | _1_ |
| **TAG 54:4 FA 20:1 (12)** | _0_ | _-1_ | _1_ | _1_ | _1_ | _0_ | _1_ | _1_ | _1_ | _1_ |
| **TAG 54:4 FA 20:2 (12)** | _1_ | _-1_ | _1_ | _1_ | _1_ | _0_ | _1_ | _1_ | _1_ | _1_ |
| **TAG 54:5 FA 20:2 (12)** | _0_ | _-1_ | _1_ | _1_ | _1_ | _0_ | _1_ | _1_ | _1_ | _1_ |
| **TAG 54:5 FA 22:4 (12)** | _1_ | _-1_ | _1_ | _1_ | _0_ | _1_ | _1_ | _1_ | _1_ | _1_ |
| **TAG 54:5 FA 22:5 (12)** | _1_ | _-1_ | _1_ | _1_ | _0_ | _0_ | _1_ | _1_ | _1_ | _1_ |
| **TAG 54:6 FA 22:5 (12)** | _1_ | _-1_ | _1_ | _1_ | _1_ | _0_ | _1_ | _1_ | _1_ | _1_ |
| **TAG 54:6 FA 22:6 (12)** | _1_ | _-1_ | _1_ | _1_ | _1_ | _1_ | _1_ | _1_ | _1_ | _0_ |
| **TAG 54:7 FA 22:5 (12)** | _0_ | _-1_ | _1_ | _1_ | _1_ | _1_ | _1_ | _1_ | _1_ | _30453_ |
| **TAG 54:7 FA 22:6 (12)** | _1_ | _0_ | _1_ | _1_ | _1_ | _1_ | _1_ | _1_ | _0_ | _0_ |
| **TAG 56:3 FA 18:0 (12)** | _0_ | _-1_ | _1_ | _1_ | _0_ | _0_ | _0_ | _0_ | _0_ | _1_ |
| **TAG 56:3 FA 20:2 (12)** | _1_ | _-1_ | _1_ | _1_ | _0_ | _0_ | _1_ | _1_ | _1_ | _1_ |
| **TAG 56:4 FA 16:0 (12)** | _0_ | _-1_ | _1_ | _1_ | _0_ | _0_ | _1_ | _1_ | _1_ | _1_ |
| **TAG 56:4 FA 18:0 (12)** | _0_ | _-1_ | _1_ | _1_ | _0_ | _0_ | _0_ | _0_ | _0_ | _1_ |
| **TAG 56:4 FA 18:1 (12)** | _0_ | _-1_ | _1_ | _1_ | _0_ | _0_ | _1_ | _0_ | _0_ | _1_ |
| **TAG 56:4 FA 20:2 (12)** | _0_ | _-1_ | _1_ | _1_ | _1_ | _0_ | _1_ | _1_ | _1_ | _1_ |
| **TAG 56:5 FA 18:0 (12)** | _0_ | _-1_ | _1_ | _1_ | _1_ | _1_ | _1_ | _1_ | _1_ | _0_ |
| **TAG 56:5 FA 20:2 (12)** | _0_ | _-1_ | _1_ | _1_ | _1_ | _0_ | _1_ | _1_ | _1_ | _1_ |
| **TAG 56:5 FA 20:5 (12)** | _0_ | _-1_ | _1_ | _1_ | _1_ | _1_ | _1_ | _0_ | _0_ | _0_ |
| **TAG 56:5 FA 22:5 (12)** | _1_ | _-1_ | _1_ | _1_ | _1_ | _1_ | _1_ | _1_ | _1_ | _0_ |
| **TAG 56:6 FA 16:0 (12)** | _0_ | _-1_ | _1_ | _1_ | _1_ | _1_ | _1_ | _1_ | _1_ | _0_ |
| **TAG 56:6 FA 18:0 (12)** | _0_ | _-1_ | _1_ | _1_ | _1_ | _1_ | _1_ | _1_ | _1_ | _0_ |
| **TAG 56:6 FA 18:1 (12)** | _0_ | _-1_ | _1_ | _1_ | _1_ | _1_ | _1_ | _1_ | _1_ | _0_ |
| **TAG 56:6 FA 18:3 (11)** | _1_ | _-1_ | _1_ | _1_ | _1_ | _1_ | _1_ | _1_ | _1_ | _0_ |
| **TAG 56:6 FA 20:2 (10)** | _0_ | _-1_ | _1_ | _1_ | _1_ | _1_ | _1_ | _1_ | _1_ | _0_ |
| **TAG 56:6 FA 22:4 (12)** | _0_ | _-1_ | _1_ | _1_ | _0_ | _0_ | _1_ | _0_ | _0_ | _1_ |
| **TAG 56:6 FA 22:5 (12)** | _1_ | _-1_ | _1_ | _1_ | _1_ | _1_ | _1_ | _1_ | _1_ | _0_ |
| **TAG 56:6 FA 22:6 (12)** | _1_ | _-1_ | _1_ | _1_ | _1_ | _1_ | _1_ | _1_ | _1_ | _0_ |
| **TAG 56:7 FA 16:0 (12)** | _0_ | _-1_ | _1_ | _1_ | _1_ | _1_ | _1_ | _1_ | _1_ | _0_ |
| **TAG 56:7 FA 16:1 (11)** | _0_ | _-1_ | _1_ | _1_ | _1_ | _1_ | _1_ | _1_ | _1_ | _0_ |
| **TAG 56:7 FA 18:1 (12)** | _0_ | _-1_ | _1_ | _1_ | _1_ | _1_ | _1_ | _1_ | _1_ | _0_ |
| **TAG 56:7 FA 20:3 (11)** | _0_ | _-1_ | _1_ | _1_ | _1_ | _1_ | _1_ | _1_ | _1_ | _0_ |
| **TAG 56:7 FA 22:5 (12)** | _1_ | _-1_ | _1_ | _1_ | _1_ | _1_ | _1_ | _1_ | _1_ | _0_ |
| **TAG 56:7 FA 22:6 (12)** | _1_ | _0_ | _1_ | _1_ | _1_ | _1_ | _1_ | _1_ | _0_ | _0_ |
| **TAG 56:8 FA 22:5 (12)** | _0_ | _-1_ | _1_ | _1_ | _1_ | _1_ | _1_ | _1_ | _1_ | _0_ |
| **TAG 58:5 FA 18:1 (11)** | _1_ | _-1_ | _1_ | _1_ | _1_ | _1_ | _1_ | _1_ | _1_ | _1_ |
| **TAG 58:6 FA 18:1 (12)** | _0_ | _-1_ | _1_ | _1_ | _1_ | _1_ | _1_ | _1_ | _1_ | _0_ |
| **TAG 58:6 FA 22:5 (12)** | _1_ | _-1_ | _1_ | _1_ | _1_ | _1_ | _1_ | _1_ | _1_ | _0_ |
| **TAG 58:7 FA 18:2 (12)** | _0_ | _-1_ | _1_ | _1_ | _1_ | _1_ | _1_ | _1_ | _1_ | _0_ |
| **TAG 58:7 FA 20:4 (10)** | _0_ | _-1_ | _1_ | _1_ | _1_ | _1_ | _1_ | _1_ | _1_ | _0_ |
| **TAG 58:7 FA 22:5 (12)** | _1_ | _-1_ | _1_ | _1_ | _1_ | _1_ | _1_ | _1_ | _1_ | _0_ |
| **TAG 58:7 FA 22:6 (12)** | _0_ | _0_ | _1_ | _1_ | _1_ | _1_ | _0_ | _1_ | _0_ | _0_ |
| **TAG 58:8 FA 22:5 (12)** | _0_ | _-1_ | _1_ | _1_ | _1_ | _1_ | _1_ | _1_ | _1_ | _0_ |

**Supplemental Table 3.** Circulating plasma concentrations of metabolites with nominally significant r-values, reported in µM. Missing values reported as NA; values below quantitation reported as BQ. Lipid values are reported in relative concentrations.

|  | **1** | **2** | **3** | **4** | **5** | **6** | **7** | **8** | **9** | **10** | **11** | **12** |
| --- | --- | --- | --- | --- | --- | --- | --- | --- | --- | --- | --- | --- |
| **Arginine** | 81.800 | 89.300 | 87.400 | 125.000 | 88.200 | 95.400 | 84.300 | 89.000 | 85.600 | 71.900 | 78.500 | 95.400 |
| **Citrulline** | 49.100 | 53.800 | 50.300 | 68.500 | 46.500 | 36.500 | 48.100 | 41.900 | 30.400 | 36.200 | 30.500 | 47.200 |
| **Glycine** | 247.000 | 234.000 | 229.000 | 325.000 | 390.000 | 163.000 | 260.000 | 289.000 | 286.000 | 146.000 | 165.000 | 258.000 |
| **Valine** | 270.000 | 226.000 | 227.000 | 197.000 | 164.000 | 280.000 | 209.000 | 208.000 | 184.000 | 347.000 | 238.000 | 203.000 |
| **Serine** | 76.600 | 58.400 | 65.000 | 61.200 | 88.100 | 85.000 | 87.400 | 88.100 | 98.600 | 62.100 | 127.000 | 83.600 |
| **D-Serine** | 1.521 | 1.798 | 1.497 | 1.927 | 1.784 | 1.835 | 3.040 | 2.207 | 2.244 | 1.904 | 2.072 | 1.458 |
| **Kynurenine** | 3.630 | 2.250 | 2.140 | 2.540 | 1.610 | 1.960 | 2.120 | 1.960 | 1.690 | 5.310 | 2.090 | 2.420 |
| **H1** | 5711.000 | 5171.000 | 4390.000 | 4721.000 | 3861.000 | 5259.000 | 5030.000 | 5155.000 | 5567.000 | 13431.000 | 4967.000 | 5547.000 |
| **SM 20:0** | 160.284 | 130.101 | 224.039 | 126.867 | 111.724 | 151.079 | 210.933 | 174.640 | 168.103 | 220.182 | 225.315 | 229.826 |
| **SM 20:1** | 23.349 | 19.552 | 30.070 | 19.073 | 17.287 | 20.897 | 24.887 | 22.406 | 23.285 | 28.731 | 31.570 | 32.382 |
| **SM 22:0** | 135.401 | 120.105 | 185.284 | 126.391 | 77.235 | 116.198 | 192.449 | 167.835 | 161.171 | 196.471 | 138.688 | 190.502 |
| **SM 22:1** | 82.526 | 69.991 | 93.141 | 69.779 | 47.880 | 66.862 | 68.024 | 81.504 | 68.428 | 104.193 | 83.551 | 90.634 |
| **SM 24:0** | 35.663 | 37.578 | 43.564 | 38.818 | 31.446 | 40.976 | 41.924 | 45.454 | 41.503 | 44.281 | 46.175 | 42.182 |
| **CE 16:0** | 382.359 | 430.806 | 344.537 | 296.415 | 283.229 | 402.435 | 659.122 | 417.538 | 406.334 | 401.243 | 450.956 | 496.977 |
| **CE 20:1** | 0.586 | 0.650 | 0.485 | 0.399 | 0.522 | 0.507 | 0.796 | 0.519 | 0.683 | . | . | 0.462 |
| **DAG 16:0/18:1** | 4.615 | 1.504 | 4.157 | 3.293 | 1.371 | 2.175 | 4.150 | 11.297 | 2.462 | 19.162 | 5.899 | 1.476 |
| **DAG 16:1/18:1** | 1.774 | 0.549 | 1.080 | 1.150 | 0.604 | 0.670 | 1.162 | 1.052 | 0.522 | 3.990 | 1.596 | 0.379 |
| **DAG 16:1/18:2** | 1.101 | 0.304 | 0.686 | 0.513 | 0.319 | 0.440 | 0.407 | 0.938 | 0.525 | 2.442 | 0.961 | 0.240 |
| **DAG 18:0/18:1** | 0.969 | 0.291 | 0.905 | 0.588 | 0.305 | 0.421 | 0.960 | 1.969 | 0.608 | 3.353 | 0.723 | 0.283 |
| **DAG 18:0/18:2** | 0.704 | 0.151 | 0.876 | 0.337 | 0.256 | 0.302 | 0.514 | 1.695 | 0.601 | 2.273 | 0.727 | . |
| **DAG 18:1/18:1** | 7.095 | 2.001 | 4.105 | 2.689 | 2.329 | 2.485 | 5.498 | 8.989 | 3.794 | 12.319 | 6.369 | 1.786 |
| **DAG 18:1/18:2** | 9.636 | 2.554 | 6.575 | 2.865 | 3.501 | 4.132 | 5.300 | 18.643 | 7.348 | 16.904 | 10.884 | 2.511 |
| **DAG 18:2/20:4** | 0.522 | 0.409 | 0.359 | 0.263 | 0.188 | 0.327 | . | 1.224 | 0.460 | . | 0.982 | 0.222 |
| **PC 16:0/16:0** | 14.400 | 18.544 | 23.017 | 11.874 | 16.264 | 14.582 | 27.743 | 18.400 | 16.454 | 22.404 | 22.907 | 27.042 |
| **PC 16:0/18:0** | 11.793 | 14.685 | 22.591 | 12.382 | 11.325 | 10.157 | 27.327 | 16.939 | 16.394 | 22.658 | 16.139 | 20.649 |
| **PC 16:0/18:1** | 202.547 | 212.432 | 200.051 | 225.579 | 152.469 | 172.505 | 371.095 | 213.962 | 165.582 | 307.858 | 249.375 | 311.397 |
| **PC 16:0/18:2** | 465.261 | 405.194 | 675.038 | 379.092 | 409.188 | 462.406 | 847.230 | 555.218 | 561.874 | 739.089 | 797.047 | 924.699 |
| **PC 16:0/20:1** | 0.684 | 0.788 | 0.722 | 0.574 | 0.672 | 0.872 | 1.191 | 1.023 | 0.894 | 1.237 | 1.348 | 0.948 |
| **PC 16:0/20:2** | 5.024 | 6.565 | 6.587 | 5.582 | 3.759 | 6.310 | 5.564 | 6.331 | 5.012 | 9.145 | 11.862 | 5.873 |
| **PC 16:0/22:5** | 26.561 | 36.310 | 26.327 | 19.688 | 12.881 | 29.108 | 51.835 | 25.887 | 29.960 | 35.634 | 28.403 | 36.600 |
| **PC 18:0/18:0** | 0.891 | 1.157 | 1.035 | 1.141 | 0.539 | 0.839 | 1.900 | 1.203 | 1.041 | 1.773 | 0.939 | 1.397 |
| **PC 18:0/18:2** | 154.861 | 148.627 | 244.218 | 148.760 | 109.256 | 139.518 | 309.396 | 216.584 | 229.538 | 280.454 | 181.123 | 320.650 |
| **PC 18:1/16:1** | 9.075 | 6.882 | 6.171 | 6.593 | 5.793 | 4.795 | 9.297 | 5.651 | 5.335 | 8.503 | 7.872 | 8.344 |
| **PC 18:2/16:1** | 11.726 | 7.638 | 12.905 | 4.781 | 8.343 | 9.141 | 13.186 | 8.826 | 8.696 | 10.258 | 13.517 | 13.451 |
| **PE 16:0/18:1** | 1.331 | 0.671 | 0.894 | 1.766 | 0.961 | 0.541 | 1.368 | 2.409 | 1.041 | 4.042 | 4.564 | 1.114 |
| **PE 16:0/18:2** | 3.767 | 0.950 | 2.830 | 3.595 | 1.989 | 1.309 | 3.602 | 4.445 | 1.903 | 9.535 | 7.797 | 3.320 |
| **PE 16:0/20:4** | 5.003 | 2.115 | 2.546 | 4.182 | 3.259 | 2.489 | 3.621 | 4.458 | 1.759 | 9.118 | 12.260 | 4.971 |
| **PE 16:0/22:6** | 9.403 | 2.237 | 3.666 | 5.200 | 6.059 | 4.000 | 19.561 | 8.983 | 7.938 | 18.994 | 30.647 | 6.394 |
| **PE 18:0/18:0** | 0.329 | 0.322 | 0.309 | 0.208 | 0.234 | 0.458 | 0.803 | 0.423 | 0.748 | 0.395 | 0.712 | 0.910 |
| **PE 18:0/18:1** | 2.494 | 1.621 | 2.224 | 2.469 | 1.423 | 1.477 | 3.324 | 5.032 | 1.878 | 5.175 | 4.568 | 2.841 |
| **PE 18:0/18:2** | 14.397 | 4.378 | 13.827 | 9.901 | 5.800 | 5.855 | 12.084 | 16.379 | 9.610 | 23.942 | 17.192 | 11.591 |
| **PE 18:0/20:4** | 21.112 | 6.939 | 10.907 | 9.956 | 10.469 | 9.806 | 10.923 | 19.463 | 8.598 | 27.685 | 32.733 | 14.386 |
| **PE 18:0/22:6** | 5.708 | 1.775 | 3.120 | 2.684 | 2.453 | 3.283 | 10.973 | 7.854 | 6.815 | 13.650 | 13.762 | 3.243 |
| **Cholesterol Esters (CE)** | 3502.168 | 3651.559 | 3397.360 | 2822.000 | 2272.249 | 3551.803 | 6085.555 | 3554.044 | 3841.386 | 3295.388 | 3677.545 | 4405.947 |
| **Diacylglycerol** | 37.498 | 13.042 | 28.796 | 20.400 | 11.951 | 17.497 | 23.793 | 68.711 | 24.707 | 95.561 | 40.440 | 11.288 |
| **Phosphatidylcholine** | 1650.967 | 1668.354 | 1896.535 | 1424.335 | 1155.758 | 1564.286 | 2690.924 | 1973.896 | 1831.096 | 2375.266 | 2435.110 | 2745.934 |
| **Phosphatidylethanolamine** | 137.591 | 150.972 | 116.527 | 109.647 | 89.388 | 96.100 | 184.561 | 170.245 | 115.554 | 198.038 | 256.047 | 202.914 |
| **Sphingomyelin** | 719.362 | 730.527 | 941.929 | 666.755 | 571.990 | 719.881 | 1073.315 | 828.705 | 782.783 | 892.120 | 851.912 | 1016.092 |
| **Triacylglycerol** | 1549.594 | 422.802 | 1227.348 | 828.558 | 410.393 | 723.025 | 816.683 | 2783.156 | 1180.253 | 4237.697 | 1512.813 | 413.211 |

**Supplemental Table 4.** Circulating CSF concentrations of metabolites with nominally significant r-values, reported in µM. Missing values reported as NA; values below quantitation reported as BQ. Lipid values are reported in relative concentrations.

|  | **1** | **2** | **3** | **4** | **5** | **6** | **7** | **8** | **9** | **10** | **11** | **12** |
| --- | --- | --- | --- | --- | --- | --- | --- | --- | --- | --- | --- | --- |
| **NAD+** | 0.010 | 0.031 | 0.015 | 0.017 | 0.014 | 0.022 | 0.010 | 0.021 | 0.012 | 0.010 | 0.011 | 0.021 |
| **Arginine** | 29.700 | 32.100 | 25.800 | 30.400 | 24.900 | 29.200 | 16.600 | 25.300 | 33.800 | 20.200 | 24.900 | 21.900 |
| **Asparagine** | 4.430* | 2.870* | 3.170* | 4.340* | 1.800* | 4.870* | BQ | 2.390* | 4.740* | 6.170 | 4.130* | 3.730* |
| **Creatinine** | 60.500 | 87.000 | 67.100 | 73.200 | 97.300 | 61.300 | 43.500 | 64.800 | 93.100 | 72.800 | 46.300 | 57.500 |
| **Glycine** | 11.000* | 11.300* | BQ | 10.800* | 15.100* | 14.000* | BQ | BQ | 12.100* | BQ | BQ | BQ |
| **D-Serine** | 0.613 | 1.082 | 1.566 | 1.185 | 2.893 | 0.823 | 0.652 | 0.833 | 1.986 | 2.053 | 1.134 | NA |
| **Diacylglycerol (DAG)** | 1.922 | 2.865 | 2.608 | 2.913 | 2.295 | 2.526 | 2.401 | 1.994 | 2.141 | 2.216 | 1.787 | 3.011 |
| **Free Fatty Acids (FFA)** | 228.107 | 231.820 | 245.039 | 205.967 | 231.851 | 218.208 | 215.737 | 213.611 | 189.653 | 206.112 | 194.006 | 209.104 |
| **DAG (16:0/16:0)** | 0.226 | 0.363 | 0.352 | 0.372 | 0.264 | 0.324 | 0.316 | 0.238 | 0.236 | 0.218 | 0.197 | 0.290 |
| **FFA 12:0** | 3.661 | 4.653 | 4.619 | 4.177 | 4.394 | 3.888 | 4.040 | 4.315 | 3.811 | 3.752 | 4.076 | 3.826 |
| **FFA 14:0** | 7.464 | 7.657 | 9.937 | 7.764 | 8.032 | 7.638 | 7.806 | 7.700 | 6.325 | 6.822 | 6.724 | 7.484 |
| **FFA 16:0** | 81.875 | 82.306 | 90.078 | 74.696 | 86.103 | 78.768 | 77.760 | 79.452 | 69.644 | 76.194 | 71.350 | 77.329 |
| **FFA 17:0** | 8.292 | 8.624 | 9.694 | 8.021 | 8.825 | 8.387 | 8.210 | 8.161 | 7.297 | 7.728 | 7.687 | 7.890 |
| **FFA 18:0** | 66.185 | 66.796 | 64.748 | 59.659 | 68.781 | 65.383 | 62.970 | 61.311 | 54.521 | 58.139 | 56.581 | 58.121 |
| **FFA 20:5** | 2.018 | 2.213 | 2.263 | 1.987 | 2.310 | 2.218 | 2.077 | 2.070 | 2.018 | 2.061 | 2.067 | 1.986 |
| **FFA 22:4** | 0.934 | 0.938 | 0.937 | 0.813 | 1.001 | 0.877 | 0.919 | 0.853 | 0.840 | 0.793 | 0.808 | 0.845 |
| **FFA22:5** | 1.034 | 1.104 | 1.034 | 0.890 | 1.082 | 0.971 | 1.008 | 0.931 | 0.942 | 0.889 | 0.788 | 0.943 |
| **TAG 36:0 FA 12:0** | 0.065 | 0.070 | 0.084 | 0.159 | 0.093 | 0.088 | 0.046 | 0.063 | 0.071 | 0.041 | 0.065 | 0.107 |
| **TAG 38:0 FA 12:0** | 0.036 | 0.040 | 0.045 | 0.088 | 0.043 | 0.048 | 0.028 | 0.040 | 0.042 | 0.030 | 0.037 | 0.052 |
| **TAG 40:0 FA 14:0** | 0.026 | 0.020 | 0.028 | 0.032 | 0.031 | 0.031 | NA | 0.024 | 0.027 | NA | 0.020 | 0.026 |
| **TAG 42:0 FA 14:0** | 0.023 | 0.025 | 0.046 | 0.049 | 0.028 | NA | 0.024 | 0.026 | 0.029 | NA | 0.017 | 0.041 |
| **TAG 48:0 FA 16:0** | 0.183 | 0.190 | 0.379 | 0.352 | 0.161 | 0.137 | 0.120 | 0.132 | 0.145 | 0.197 | 0.101 | 0.310 |
| **TAG 48:0 FA 18:0** | 0.028 | 0.031 | 0.058 | 0.051 | 0.030 | 0.027 | NA | 0.022 | 0.023 | 0.024 | 0.022 | 0.039 |
| **TAG 49:0 FA 17:0** | 0.044 | 0.033 | 0.065 | 0.074 | 0.038 | 0.033 | 0.030 | 0.035 | 0.035 | 0.038 | 0.022 | 0.069 |
| **TAG 52:0 FA 18:0** | 0.123 | 0.183 | 0.124 | 0.129 | 0.130 | 0.126 | 0.105 | 0.093 | 0.104 | 0.116 | 0.105 | 0.108 |
| **PC 16:0/16:0** | 0.709 | 0.977 | 0.788 | 0.666 | 0.789 | 0.899 | 0.270 | 0.335 | 0.513 | 0.476 | 0.341 | 0.789 |
| **PC 16:0/18:1** | 2.796 | 4.281 | 2.665 | 3.305 | 4.021 | 3.148 | 1.647 | 1.769 | 2.805 | 2.928 | 2.548 | 3.522 |
| **SM 14:0** | 0.058 | 0.085 | 0.055 | 0.070 | 0.070 | 0.072 | 0.037 | 0.042 | 0.057 | 0.066 | 0.049 | 0.072 |
| **SM 16:0** | 0.765 | 1.309 | 0.602 | 0.694 | 0.918 | 0.777 | 0.320 | 0.422 | 0.718 | 0.673 | 0.587 | 0.561 |
| **SM 18:0** | 0.604 | 1.066 | 0.623 | 0.728 | 0.877 | 0.988 | 0.338 | 0.351 | 0.578 | 0.596 | 0.463 | 0.767 |
| **SM 18:1** | 0.116 | 0.170 | 0.088 | 0.103 | 0.122 | 0.120 | 0.046 | 0.052 | 0.097 | 0.081 | 0.081 | 0.109 |
| **SM 20:1** | 0.064 | 0.094 | 0.052 | 0.054 | 0.062 | 0.059 | 0.039 | NA | 0.045 | 0.051 | 0.052 | 0.049 |
| **Phosphatidylcholine (PC)** | 5.676 | 9.480 | 5.195 | 5.757 | 6.893 | 5.277 | 2.234 | 2.691 | 4.651 | 4.830 | 4.880 | 6.283 |

***** Several concentrations at the limit of quantitation.
